# HDLs extract lipophilic drugs from cells

**DOI:** 10.1242/jcs.258644

**Published:** 2022-01-31

**Authors:** Adi Zheng, Gilles Dubuis, Maria Georgieva, Carla Susana Mendes Ferreira, Marc Serulla, Maria del Carmen Conde Rubio, Evgeniya Trofimenko, Thomas Mercier, Laurent Decosterd, Christian Widmann

**Affiliations:** 1 Department of Biomedical Sciences, University of Lausanne, Bugnon 7, 1005 Lausanne, Switzerland; 2 Laboratory of Clinical Pharmacology, Lausanne University Hospital (CHUV) and University of Lausanne, 1011 Lausanne, Switzerland

**Keywords:** High-density lipoprotein, HDL, Cell death, Thapsigargin, Glibenclamide, Staurosporine, Doxorubicin, Rhodamine 123, Drug efflux

## Abstract

High-density lipoproteins (HDLs) prevent cell death induced by a variety of cytotoxic drugs. The underlying mechanisms are however still poorly understood. Here, we present evidence that HDLs efficiently protect cells against thapsigargin (TG), a sarco/endoplasmic reticulum (ER) Ca^2+^-ATPase (SERCA) inhibitor, by extracting the drug from cells. Drug efflux could also be triggered to some extent by low-density lipoproteins and serum. HDLs did not reverse the non-lethal mild ER stress response induced by low TG concentrations or by SERCA knockdown, but HDLs inhibited the toxic SERCA-independent effects mediated by high TG concentrations. HDLs could extract other lipophilic compounds, but not hydrophilic substances. This work shows that HDLs utilize their capacity of loading themselves with lipophilic compounds, akin to their ability to extract cellular cholesterol, to reduce the cell content of hydrophobic drugs. This can be beneficial if lipophilic xenobiotics are toxic but may be detrimental to the therapeutic benefit of lipophilic drugs such as glibenclamide.

## Abstract (precis)

**Summary:** HDLs, akin to their capacity for extracting cholesterol, can remove lipophilic compounds from cells, thus protecting the cells when these compounds are toxic.

## INTRODUCTION

High-density lipoproteins (HDLs) possess multifaceted biological protective properties, including anti-inflammation, anti-oxidation and anti-apoptosis, which can be beneficial for the treatment of diseases (Gordon et al., 2011). The mechanism(s) used by HDLs to protect cells are poorly understood (von Eckardstein and Widmann, 2014). Sphingosine-1-phosphate receptor-mediated Akt signaling has been proposed to be a pathway triggered by HDLs that protects cells (Nofer et al., 2004). However, a recent study (Zheng et al., 2019b) found no evidence for Akt being involved in HDL-mediated cell protection. How HDLs protect cells remains therefore largely unexplained.

HDLs inhibit endoplasmic reticulum (ER) stressor-induced death (von Eckardstein and Widmann, 2014). They do so via different mechanisms depending on which ER stressors are used. For example, in β-cells, HDLs block the ability of the sarco/endoplasmic reticulum Ca^2+^-ATPase (SERCA) pump inhibitor thapsigargin (TG) from inducing a strong unfolded protein response (UPR) and subsequently cell death (Pétremand et al., 2012), but HDLs did not prevent the inhibitory action of TG on SERCA (Pétremand et al., 2012). On the other hand, while HDLs protected β-cells from the protein glycosylation inhibitor tunicamycin, it did so with minimal impact on the UPR (Puyal et al., 2013). Hence, in one case HDL-mediated protection was associated with UPR inhibition while in the other, protection occurred despite activation of the UPR. This indicates that HDLs can activate more than one anti-death pathway in cells.

A well-known property of HDLs is their capacity to extract cholesterol from cells. HDLs can also bind to drugs, especially hydrophobic molecules (Sobansky and Hage, 2012) and act as drug carriers (Mooberry et al., 2010; Raut et al., 2018). Whether the binding capacity of HDLs to particular drugs translates into an ability to extract the drugs from cells has not been tested yet. This prompted us to investigate whether HDLs can extract drugs from cells and whether this protects cells from drugs with cytotoxic properties.

## RESULTS

### The effect of HDLs on different TG concentrations

**Fig. 1.**
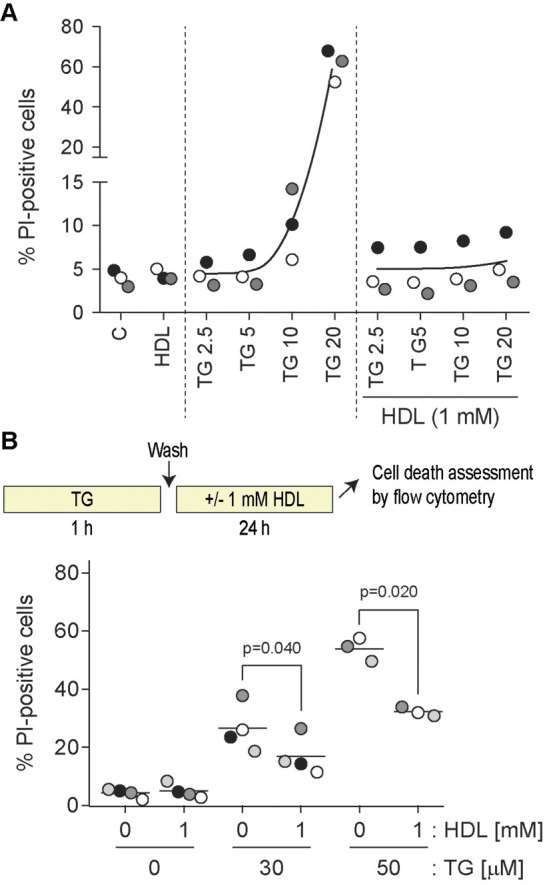
**HDL-mediated inhibition of TG-induced cell death.** (A) DLD-1 cells were seeded in six-well plates (100,000 per well). After 24 h, cells were treated for 48 h with the indicated concentrations (in µM) of TG in the presence or in the absence of HDLs. Cell death was measured by flow cytometry following PI staining. Symbols with a given shade of gray are derived from a given independent experiment. The black curves go through the average values of the different experiments. (B) DLD-1 cells were seeded in six-well plates (100,000 per well). After 24 h, cells were pre-treated 1 h with the indicated concentrations of TG (in µM). Cells were washed once with PBS and then incubated or not with 1 mM HDLs for an additional 24 h period at which time cell death was assessed by PI incorporation using flow cytometry. *P*-values were calculated using a one-way ANOVA with a post-hoc Dunnett's multiple comparison test.

**Fig. 2.**
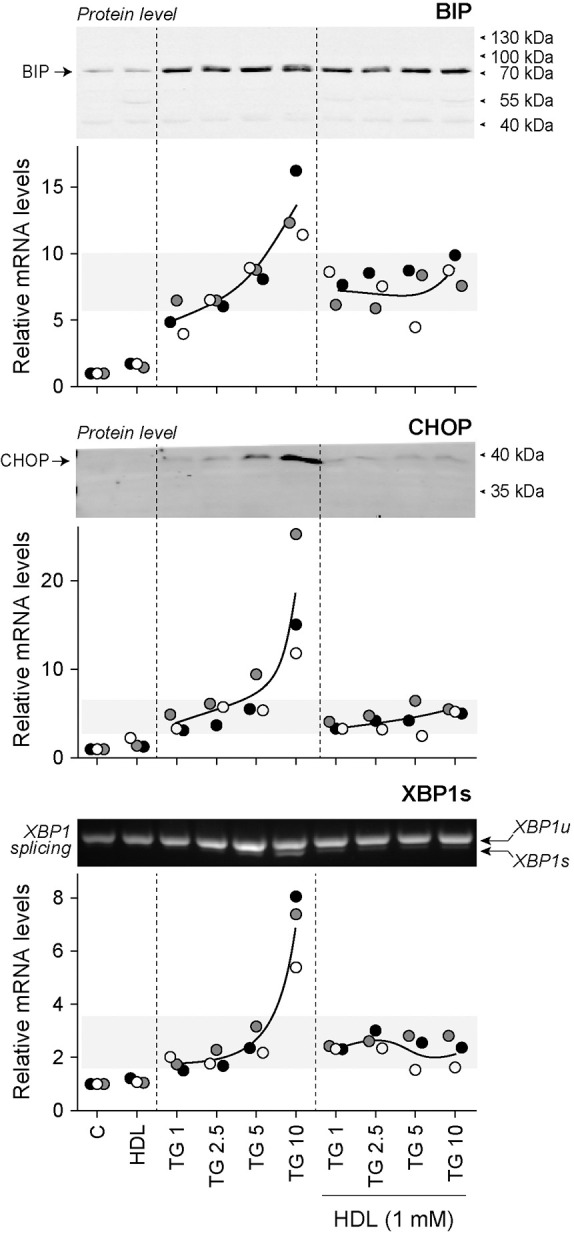
**HDLs inhibit ER stress marker expression induced by high TG concentrations.** DLD-1 cells were seeded in 6-well plates (200,000 per well). After 24 h, cells were treated for 24 h with the indicated concentrations (in µM) of TG in the presence or in the absence of HDLs. The mRNA levels of UPR proteins (BIP, CHOP and XBP1s) were measured by qRT-PCR. The corresponding BIP and CHOP protein levels assessed by western blotting are shown above the graphs. The extent of XBP1 splicing is presented above the XBP1s mRNA quantitation graph. The RT-PCR data are from three independent experiments (each labeled with different symbols). Western blots and XBP1 splicing assessment were performed two or three times with similar results (only one representative blot is shown). XBP1u, unspliced XBP1 mRNA; XBP1s, spliced XBP1 mRNA.

TG is a specific and irreversible inhibitor of the SERCA pump (Doutheil et al., 1999; Michelangeli and East, 2011). SERCA deficiency induces ER dysfunction (Tong et al., 2016). As shown previously (Zheng et al., 2019b), HDLs were able to protect cells from high concentration (≥10 µM) TG-induced death (Fig. 1A), even when added after cells were pre-loaded with TG (Fig. 1B). TG induced the mRNA expression of ER stress-related markers [BIP, CHOP and XBP1s (BIP and CHOP are also known as HSPA5 and DDIT3, respectively; XBP1s refers to the spliced isoform of XBP1)] in a concentration-dependent manner (Fig. 2). HDLs decreased the mRNA expression of these genes in cells stimulated with high TG concentrations (10 μM) to the levels detected when lower TG concentrations (≤5 μM) were used alone. However, HDLs did not alter BIP, CHOP and XBP1s mRNA expression induced by TG concentrations ≤5 μM. This was also seen when CHOP protein levels or XBP1 splicing were assessed; HDLs inhibited CHOP expression and XBP1 splicing induced by high TG concentrations to levels seen in cells stimulated with low TG concentrations. As for the corresponding mRNA, HDLs did not affect CHOP protein expression or the extent of XBP1 splicing induced by low TG concentrations. At the protein level, BIP was already maximally induced by low TG concentrations (i.e. there was no further increase when higher concentrations of TG were used). HDLs did not reduce the expression of BIP protein induced by TG, which would be expected if BIP levels were already reaching a plateau at low TG concentrations.

### The effect of HDL on SERCA2 knockdown-induced ER stress

**Fig. 3.**
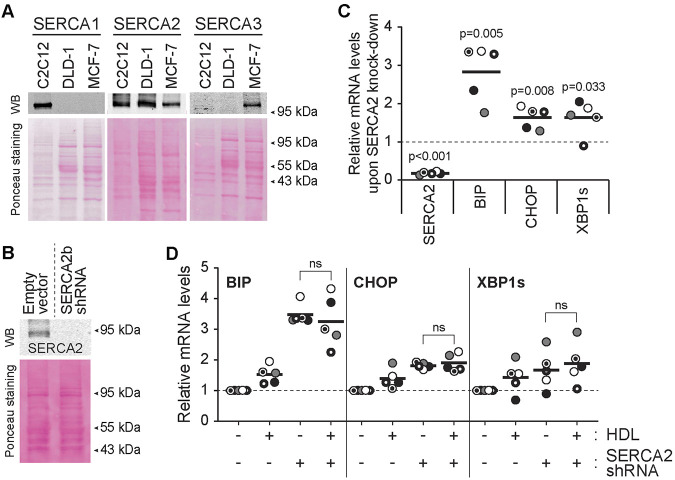
**HDLs do not alleviate the stress response mediated by SERCA knockdown.** (A) SERCA1, SERCA2 and SERCA3 expression in DLD-1 cells was assessed by western blotting. C2C12 and MCF-7 cell lysates were used as positive controls for SERCA1 and SERCA3, respectively. (B) Knockdown efficiency of SERCA2 in DLD-1 cells. (C) The mRNA expression of SERCA2 and ER stress markers (BIP, CHOP and XBP1s) in SERCA2-knockdown cells. (D) Effect of HDLs on the induction of ER stress marker mRNA expression induced by SERCA2 knockdown. In panels C and D, the results are normalized to the expression values obtained in untreated cells (dashed line). Symbols with a given shape and shading are derived from a given independent experiment. The black lines correspond to the mean. *P*-values were calculated with a two-tailed paired *t*-test; ns, not significant.

TG is a long-lasting irreversible inhibitor of SERCA but cells can adapt to this inhibition and restore ER homeostasis and protein translation and become refractory to the TG effect (Mengesdorf et al., 2001). The observation provided in Fig. 2 indicates that HDLs do not alter the UPR response induced by concentrations of TG below 5 µM, which are known to fully inactivate SERCA (Michelangeli and East, 2011). HDLs should therefore not affect the UPR response induced by SERCA invalidation. To assess this point, we knocked down SERCA2 in DLD-1 cells (SERCA1 and SERCA3 are not expressed in this cell line; Fig. 3A). SERCA2 knockdown (Fig. 3B) in DLD-1 induced mild ER stress, as indicated by the upregulation of ER stress markers (Fig. 3C) to levels equal or lower than those obtained with low concentrations of TG (compare Fig. 2 and Fig. 3). HDLs were not able to suppress the ER stress response induced by SERCA2 knockdown (Fig. 3D). By themselves, HDLs slightly stimulated the expression of UPR genes, a likely consequence of their ability to modulate cholesterol levels in cells (Zhang et al., 2018). Despite their inability to prevent a SERCA2 knockdown-induced mild ER stress response (Fig. 3), HDLs were still capable of blocking the strong UPR response induced by high concentrations of TG in SERCA2 knockdown cells (Fig. S1).

We have previously shown that HDLs could not prevent the transient increase of cytosolic Ca^2+^ induced by the inhibitory action on SERCA (Pétremand et al., 2012). Coupled with the present results, we can conclude that HDLs inhibit the SERCA-independent UPR and lethality induced by high concentrations of TG but that HDLs neither interfere with the ability of TG to inhibit SERCA nor with the subsequent ER mild stress response that ensues.

### HDLs extract lipophilic drugs from cells

**Fig. 4.**
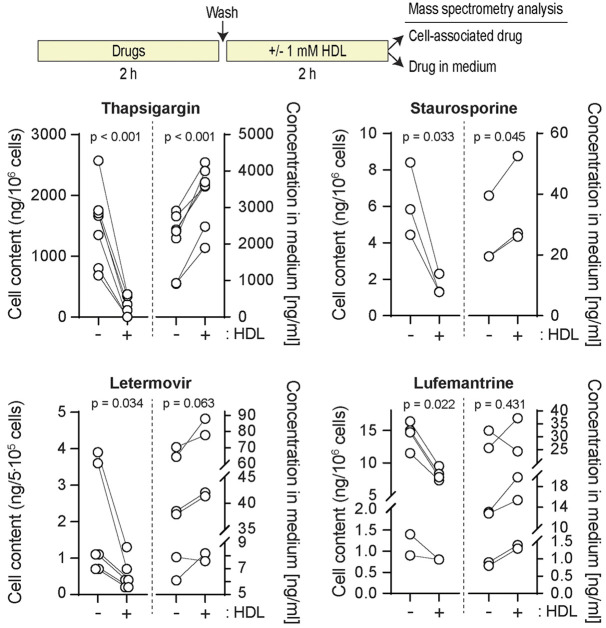
**HDLs extract hydrophobic drugs from cells.** DLD1 cells (500,000 cells) plated in six-well plates were treated 24 h later as depicted in the scheme above the figure (also see Materials and Methods) using the following concentrations: thapsigargin, 20 µM; staurosporine, 100 nM; letermovir and lumefantrine, 1 µg/ml. The cell-associated drug content and the drug found in the medium were then quantitated as described in the Materials and Methods. The data correspond to 4–7 replicates coming from two or three independent experiments. For the letermovir and lufemantrine graph, two independent experiments (two replicates each) were performed several weeks apart using the same drug stocks. Drug degradation may account for the lower signal obtained for the last two replicates. *P*-values were calculated with a two-tailed paired *t*-test.

**Fig. 5.**
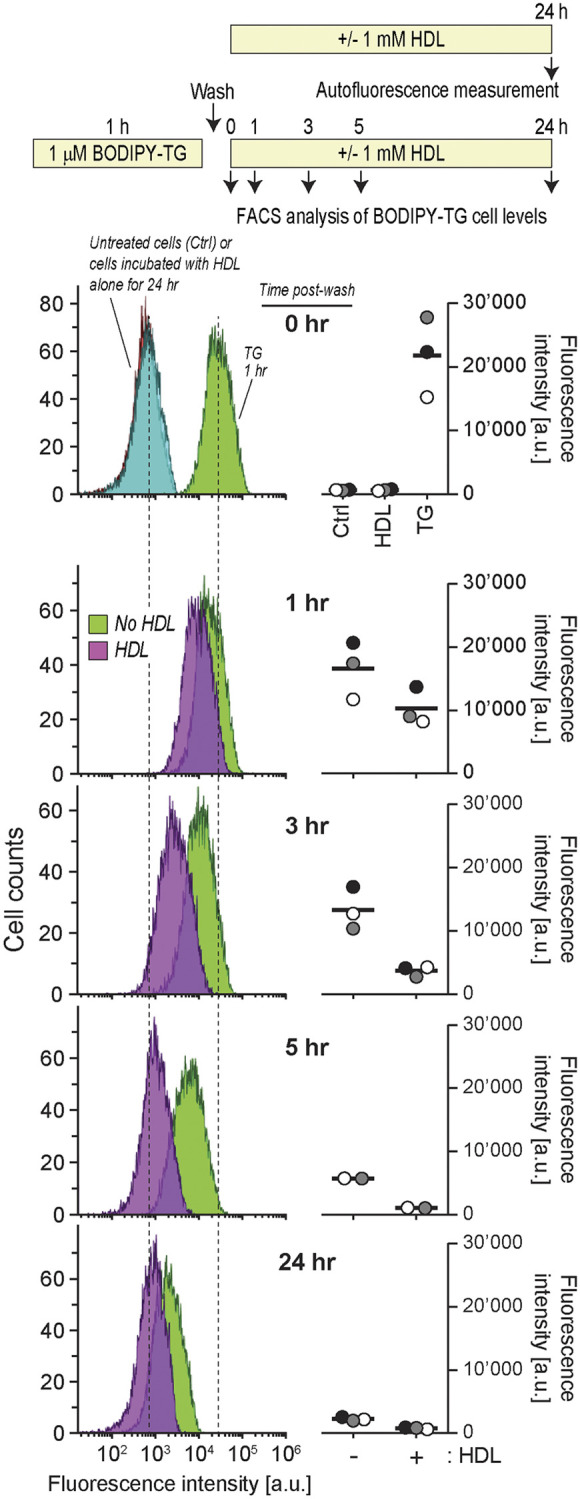
**Kinetics of spontaneous and HDL-mediated TG cell release.** Min6 cells (300,000 cells per well) were seeded in six-well plates and cultured overnight. After treatment with 1 μM BODIPY–TG for 1 h, cells were washed with PBS once and then incubated with medium or HDLs for the indicated periods of time before their drug content was analyzed by flow cytometry. In the ‘0 hr’ panel, the autofluorescence of untreated cells and cells incubated with HDLs alone for 24 h are also shown (the two corresponding distributions fully overlap), as well as the profile of TG–BODIPY-incubated cells right after the washing step. The peak autofluorescence of the cells and the peak fluorescence intensity of 1 h TG–BODIPY-incubated cells are indicated by dashed lines. The quantification of two or three independent experiments (labeled with different symbols) is depicted on the right-hand side of the flow cytometry graphs. The latter are derived from the experiment corresponding to the gray symbols. The bars represent the mean of the cytometry distribution profiles. a.u., arbitrary units.

**Fig. 6.**
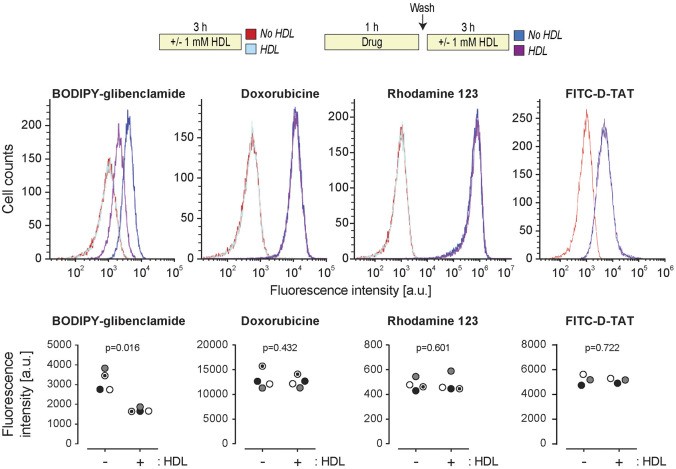
**Effect of HDLs on BODIPY-glibenclamide, doxorubicin hydrochloride, rhodamine 123 and FITC-D-TAT efflux.** HeLa cells (120,000 per well) were plated in six-well plates. Then cells were treated as indicated in the scheme above the graphs (drug concentrations: BODIPY-glibenclamide, 1 μM; doxorubicine, 10 μM; Rhodamine 123, 5 μM; FITC-D-TAT, 10 μM). Drug-associated fluorescence in cells was assessed by flow cytometry. The quantification (mean of the cytometry distribution profiles) of three or four independent experiments (labeled with different symbols) is depicted below the flow cytometry profiles. *P*-values were calculated with a two-tailed paired *t*-test. a.u., arbitrary units.

A classical role of HDLs is to facilitate cholesterol efflux and to remove excess cholesterol from cells (Gordon et al., 2011). Conceivably, HDLs might have the capacity to decrease lipophilic drug content inside cells and therefore protect them against their lethal effects. To test this hypothesis, we determined whether HDLs could modulate the intracellular TG content. Fig. 4 shows that HDLs decreased the amount of cell-associated TG and this was paralleled with an increase of TG in the HDL-containing medium. The ability of HDLs to extract TG from cells was also evidenced using a fluorescent version of TG (BODIPY–TG) (Fig. 5; Fig. S2). The ability of HDLs to reduce the TG cellular load was not a consequence of HDLs modulating the expression of SERCA (Fig. S3). HDLs were capable of extracting other lipophilic drugs, such as staurosporine, letermovir, lufemantrine (Fig. 4) and glibenclamide (Fig. 6). In contrast to lipophilic drugs, hydrophilic drugs or compounds like doxorubicin hydrochloride, an anticancer drug, or Rhodamine 123, an ABCB1/p-glycoprotein activity sensor, and FITC-D-TAT were not extracted from cells by HDLs (Fig. 6). Analyzing the physicochemical properties of the drugs used here (plus cholesterol as a classical HDL interactor) using the SwissADME web tool (Daina et al., 2017) substantiated the notion that HDLs have the capacity to bind lipophilic but not hydrophilic molecules (Fig. S4).

### Capacity of serum components other than HDLs to extract TG from cells

**Fig. 7.**
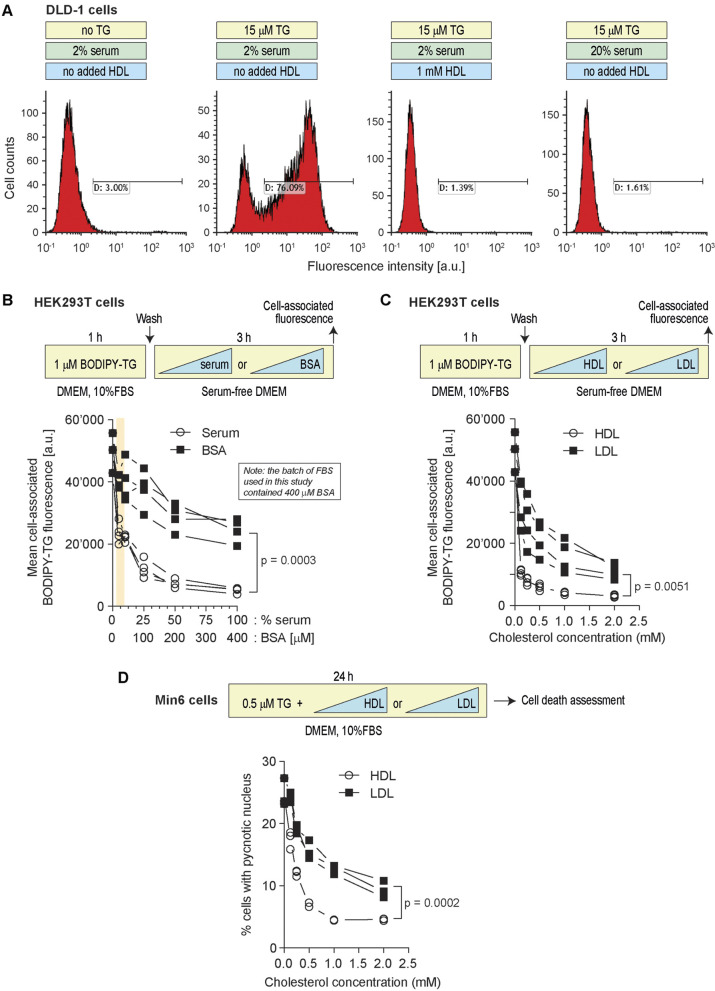
**Serum components other than HDLs can extract TG from cells but less efficiently than HDLs.** (A) DLD-1 cells (100,000 cells per well) were treated as indicated in the scheme above the panel. Cell death (%) was measured by PI staining (section indicated with D). (B) HEK293T cells (150,000 cells per well) were treated first 1 h with 1 µM BODIPY–TG in DMEM with 10% FBS and then 3 h with increasing concentrations of serum or BSA in serum-free DMEM. The concentrations of BSA were chosen as to correspond to those found in serum (i.e. 0.4 mM BSA in 100% serum). Cell-associated BODIPY–TG levels were assessed by flow cytometry. (C) Alternatively, instead of being incubated with serum or BSA, HEK293T cells were treated with increasing concentrations of HDLs or LDLs. The data are represented based on cholesterol content. (D) Min6 cells (300,000 cells per well) were seeded in six-well plates for 24 h. Then cells were treated with 0.5 µM TG in the presence of increasing concentrations of the indicated lipoproteins for 24 h. Cell death was assessed by determining the percentage of cells with pycnotic nuclei. *P*-values were calculated with a two-way ANOVA. a.u., arbitrary units.

Lipoproteins, as well as proteins with the ability to bind lipophilic compounds, such as albumin (Baker, 1998), are present in the serum used to supplement culture media. Conceivably, increasing the serum concentration in the media could negatively impact the ability of TG to accumulate in cells. This is indeed what was observed in two different cell lines (Fig. 7A,B). Albumin alone was able to extract TG from cells but this accounted for only a fraction of what serum could achieve (Fig. 7B). For example, there was ∼50% less TG in cells incubated with 5% serum compared to cells incubated in the absence of serum but the decrease was only ∼10% when the concentration of bovine serum albumin (BSA) found in 5% serum was used (20 µM; see tan region in Fig. 7B). LDLs were also able to extract fluorescent TG from cells but again less efficiently than HDLs (Fig. 7C). Moreover, HDLs were also more potent than LDLs at protecting cells from the deleterious action of TG (Fig. 7D). Altogether, these results indicate that there are several serum components that can bind to lipophilic drugs such as TG but that none are as potent as HDLs to do so. The dose response curves obtained with serum or with the corresponding HDL concentrations are similar (compare the open circle curves in B and C of Fig. 7). This indicates that HDLs explain most of the TG cell extracting activity of serum.

### ABCB1 involvement in HDL-mediated drug efflux is cell type specific

To further elucidate the molecular mechanism of drug export by HDLs, we examined the role of several transporters or receptors that have been related to cholesterol transport (Litvinov et al., 2018). SR-BI (also known as SCARB1) is a surface protein HDLs can dock to (Trigatti et al., 2003) and that may therefore participate in drug efflux to these lipoproteins. We tested this hypothesis using SR-BI-knockout cells (Fig. S5A). These cells have the same capacity as control cells to take up BODIPY–TG (Fig. S5B). HDL-mediated BODIPY–TG efflux was not altered by the absence of SR-BI (Fig. S5C), a finding consistent with the observation that SR-BI gene invalidation does not prevent HDLs from protecting cells against TG (Pétremand et al., 2012). These results suggest that SR-BI plays no role in the ability of HDLs to extract lipophilic drugs from cells.

**Fig. 8.**
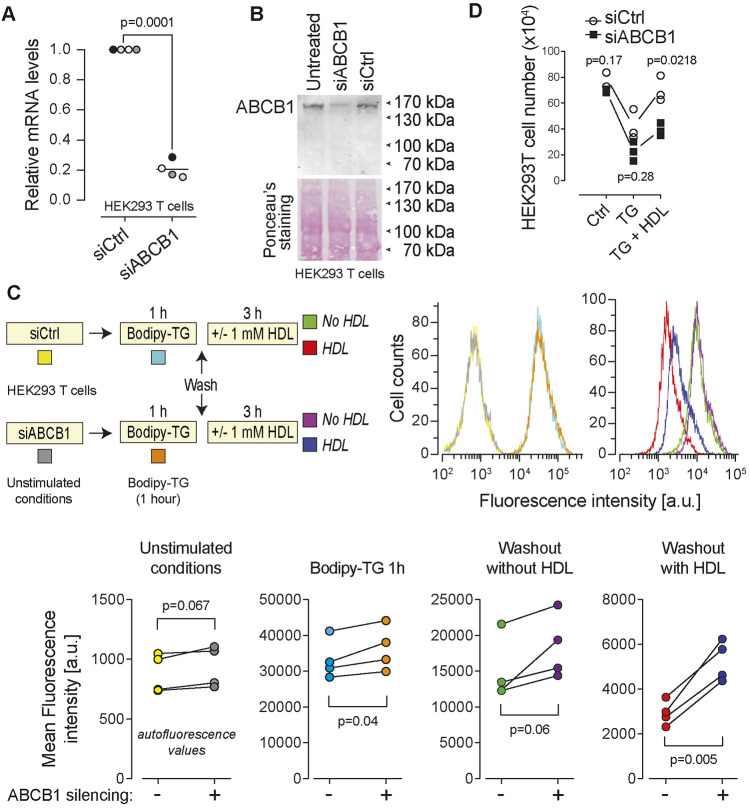
**ABCB1 participates in HDL-mediated BODIPY-TG efflux.** HEK293T cells (80,000 per well of 12-well plates) were cultured overnight and then transfected with a control siRNA pool (siCtrl) that does not target any human mRNAs and a siRNA pool directed at ABCB1 (siABCB1). The cells were analyzed 72 h post-transfection. (A,B) Knockdown efficiency at the RNA level was evaluated by RT-PCR (A) and at the protein level by western blotting (B). (C) Control and ABCB1-silenced cells were incubated with 1 μM BODIPY–TG and HDLs as indicated in the scheme. Cell-associated fluorescence was then assessed by flow cytometry. The quantification of four independent experiments is depicted below the flow cytometry profiles. (D) Alternatively, at 48 h post transfection, the cells were treated with 20 µM TG for 24 h in the absence or in the presence of 1 mM HDLs. Cells remaining attached to the plates (viable cells) were counted using light microscopy. *P*-values were calculated with a two-tailed paired *t*-test. a.u., arbitrary units.

The ABCA1, ABCG1, ABCB1 and ABCG2 transporters can protect cells from the toxic effect of endogenous, as well as xenobiotic molecules, by mediating their efflux from cells (Glavinas et al., 2004; Storti et al., 2019; Toyoda et al., 2019). We aimed to use siRNA-mediated silencing to test their involvement in HDL-mediated drug efflux. Western blot analysis revealed that ABCA1, ABCB1 and ABCG2 are differentially expressed in MCF7, HEK293T, HeLa, HCT116, and DLD-1 cells (Fig. S6A). The antibodies directed against ABCG1 (Table S1) were found to be non-specific (i.e. the band seen in Fig. S6A was still detected by these antibodies in ABCG1 knockout cells; data not shown). The expression of this transporter can therefore not be evaluated by western blotting. The silencing efficiency for ABCA1 (∼50% knockdown) was too weak, preventing us from testing its involvement in HDL-mediated drug efflux. On the other hand, ABCB1 and ABCG2 silencing was relatively efficient (Fig. 8A,B; Fig. S6B). ABCB1 knockdown partially inhibited the ability of the HDL to promote BODIPY–TG efflux in HEK293T cells (Fig. 8C) and impaired the capacity of HDLs to protect the cells from TG-induced death (Fig. 8D). In the absence of HDLs, TG-induced death was also exacerbated by ABCB1 silencing, a likely consequence of lipoproteins in the serum being less able to extract the drug from cells (Fig. 8D). In contrast to what was observed for ABCB1, ABCG2 silencing had no impact on the drug efflux capacity of HDLs (Fig. S6C). To further evaluate the role of ABC transporters in HDL-mediated drug efflux, we disrupted the ABCB1 and ABCG1 genes using the CRISPR/Cas9 technology in DLD-1 cells (Fig. S7A,B). The DLD-1 cell line does not seem to express the ABCA1 and ABCG2 transporters (see Fig. S6A). Hence ABCB1/ABCG1 double knockout DLD-1 cells do not express four ABC transporters that have the potential to mediate drug efflux. Fig. S7C shows that TG was still efficiently extracted by HDLs in ABCB1/ABCG1 double knockout DLD1 cells. This indicates that ABC transporters are not mandatory or required for the process of HDL-mediated drug extraction. They can however favor this process in some cases (e.g. ABCB1 in HEK293T cells; see Fig. 8).

## DISCUSSION

Low HDL levels are associated with the risk of developing several diseases including cardiovascular disease and diabetes (Barter et al., 2007; Drew et al., 2012; Navab et al., 2011; von Eckardstein and Widmann, 2014). Whether high HDL levels directly participate in protective responses or whether they are mere markers of healthier metabolic states is a debated issue (Haase et al., 2015; Jomard and Osto, 2020; von Eckardstein and Rohrer, 2016). What is clear, however, is that HDLs can protect cells from a variety of toxic compounds (von Eckardstein and Widmann, 2014). Earlier work has suggested that HDLs activate intracellular pathways, such as those leading to Akt activation (Nofer et al., 2001), to protect cells, but this has recently been challenged (Zheng et al., 2019b). The mechanisms allowing HDLs to protect cells are therefore still debated and require further characterization. The present work provides evidence that the drug efflux capacity of HDLs contributes to their protective effects against lipophilic drugs such as TG and staurosporine.

We previously showed that HDLs blocked the ER stress response induced by TG in pancreatic β-cells, efficiently protecting the cells from undergoing apoptosis (Pétremand et al., 2012). HDLs were also shown to protect β-cells from tunicamycin, another ER stressor (Puyal et al., 2013). This protection occurred downstream of the ER stress response, as HDLs in this case did not interfere with the capacity of tunicamycin to induce an ER stress response (Puyal et al., 2013). TG is lipophilic and, as shown in the present study, is readily extracted from cells by HDLs. HDLs therefore protect cells from TG by reducing the exposure of the cells to this drug. In contrast, tunicamycin is hydrophilic and is consequently not expected to be captured by HDLs. This can explain why HDLs do not prevent tunicamycin from activating an ER stress response. The way HDLs are inhibiting the death response induced by tunicamycin must therefore occur downstream of the activation of the ER stress response but how this occurs has eluded characterization so far.

Our data indicate that HDLs do not affect the ability of TG to inhibit SERCA and the low ER stress response that ensues. This can be explained by the fact that very low concentrations of TG inhibit SERCA in an irreversible manner (Ki values of 0.2, 1.3, and 12 nM for SERCA1b, SERCA2b, and SERCA3a, respectively; Wootton and Michelangeli, 2006). Hence, even though HDLs can remove a substantial amount of TG from cells (Fig. 4), the concentration of TG that remains in cells is likely still sufficient to fully inhibit the SERCA pumps. On the other hand, the toxic effects mediated by high (≥10 µM) TG concentrations can be alleviated by HDLs, presumably because HDLs remove enough TG from cells so that the remaining cellular concentrations of TG are now below the lethal threshold of the drug. Consequently, the SERCA-independent toxic effects of TG appear reversible. Evidence has been presented that these toxic effects are a consequence of mitochondrial dysfunction resulting from the ability of TG to induce mitochondrial permeability transition (Korge and Weiss, 1999).

HDLs are well-known cholesterol carriers. They can extract cholesterol from certain tissues (e.g. atherosclerotic plaques) but they can also deliver this lipid to other tissues, such as steroidogenic organs (e.g. adrenal glands, gonads). In some species, such as the mouse, HDLs are the main cholesterol carriers (in humans triglyceride-rich lipoproteins are the main cholesterol carriers) (Dietschy and Turley, 2002; van Ree et al., 1995). The capacity of HDLs to take up or deliver cholesterol depends on how much cholesterol they already carry and the concentration of cholesterol found in membranes of the cells they interact with. By extension, whether HDLs give to or take from cells lipophilic drugs will depend on the respective levels of the drugs in the lipoprotein particles and in the plasma membrane. The capacity of HDLs to carry drugs has recently been discussed in the context of drug delivery (Chaudhary et al., 2019). HDLs can be viewed as natural nanoparticles that can be loaded with specific cargos bearing therapeutic properties (Ma et al., 2018). Based on the finding reported in the present study, the drug efflux capacity of HDLs should be included in the design of strategies based on delivering therapeutic compounds to cells using HDL carriers. To illustrate this point, we can mention the observation that paclitaxel brought into cells by paclitaxel-loaded recombinant HDL particles is reduced by 70% by free HDL particles (Mooberry et al., 2010). Consequently, when lipoproteins are utilized as drug carriers, drug dosage should be optimized to balance the effect of efflux to plasma HDLs.

The present work shows that HDLs are a potent ‘extractor’ of lipophilic compounds but are poor at promoting the efflux of hydrophilic drugs from cells (Fig. 6). Hydrophilic drugs may therefore represent more ideal cargos to be transported by lipoproteins, as long as they can be loaded on lipoprotein particles (Yuan et al., 2013).

HDLs can extract toxic xenobiotics such as TG, but can also promote the cellular efflux of therapeutic compounds such as the glibenclamide anti-diabetic drug (Fig. 6). Determining the intrinsic capacity of HDLs from patients to capture a given drug may therefore inform clinicians on which drug to use, and at which dosage, for optimal therapeutic treatment.

The likely fate of HDL-extracted drugs in humans is that they are carried to the liver by HDLs. Scavenger receptors such as SR-BI expressed by hepatocytes can then bind HDLs carrying the extracted drugs allowing the drugs to be taken up by the cells. In hepatocytes, the drugs may be inactivated and excreted in the bile (Almazroo et al., 2017; Ouimet et al., 2019).

Our data indicate that the ability to promote lipophilic drug efflux is not a unique specificity of HDLs, but is also present in two other serum components, albumin and LDLs. Albumin can enhance cellular cholesterol efflux through an aqueous diffusion mechanism (Sankaranarayanan et al., 2013). Albumin may employ a similar mechanism to promote drug efflux, but we show here in the case of TG that this is far less efficacious than what can be achieved by lipoproteins (Fig. 7). LDLs also contribute to the ability of serum to promote TG efflux from cells but they are less potent than HDLs to do so (Fig. 7). These results highlight the importance of controlling the impact of serum in experiments assessing the cellular activity of drugs and compounds, in particular those that are lipophilic.

Cholesterol efflux to HDLs is promoted by a series of ABC transporters. For example ABCA1 is involved in cholesterol transfer to nascent HDL particles and ABCG1 mediates the HDL cholesterol loading during the process of reverse cholesterol transport from arteries to the liver (Cavelier et al., 2006). It could have been anticipated that the way HDLs mediate drug and cholesterol efflux was similar, and consequently that HDL-mediated drug extraction can be mediated (or at least favored) by specific ABC transporters. This would be consistent with previous reports indicating that ABCA1, ABCG1, ABCB1 and ABCG2 transporters protect cells from the toxic effect of endogenous as well as xenobiotic molecules by mediating their efflux from cells (Glavinas et al., 2004; Storti et al., 2019; Toyoda et al., 2019). Our results indicate however that these four ABC transporters are dispensable for efficient HDL-mediated TG extraction in the DLD1 cell line (Fig. S7). Only in HEK293T cells could we show an impact on the efficiency of HDL-mediated TG extraction when the ABCB1 transporter was knocked down (Fig. 8). Even in this case, TG efflux was not solely dependent on ABCB1 as TG efflux still occurred with delayed kinetics in its absence. Even though specific ABC transporters may favor HDL-mediated drug extraction in some cell lines, it can be concluded that ABC transporters are not necessarily required for efficient HDL-mediated drug efflux.

Clinical investigation is still needed to understand whether drug delivery to tissues and cells are causally associated or not with specific lipoprotein levels. Further research will have to be performed to evaluate *in vivo* how and to what extent HDLs extract hydrophobic compound and xenobiotics. This will expand our knowledge on the transport functions of lipoproteins beyond their classical physiological lipid-binding capacity.

## MATERIALS AND METHODS

### Reagents

Lipofectamine RNAiMAX transfection reagent (ref. 13778030) was from Thermo Fisher Scientific. BODIPY-FL TG and BODIPY-FL Glibenclamide were purchased from Marker Gene and Life Technology (ref. M4700, lot 291AAN029; ref. E34251, lot 2069641, respectively). Hoechst 33342 was from Thermo Fisher Scientific (ref. H3570). Doxorubicin hydrochloride was from Sigma-Aldrich (ref. 44583-1MG), Rhodamine 123 was purchased from Sigma-Aldrich (ref. 83702-10MG). The siPOOLs directed at ABCA1 (lot 19-1-001), ABCB1 (lot 5243-1-001), ABCG1 (lot 9619-1-001) and ABCG2 (lot 9429-1-001), and a non-specific control siPOOL (lot N000-051), were purchased from Biotech. The siPOOLs are high-complexity pools of 30 optimally designed siRNAs (Hannus et al., 2014). Letermovir (MSD Merck Sharp & Dohme AG, Lucerne, Switzerland; catalog number J05AX18, lot T029327) and Lumefantrine (Toronto Research Chemicals, Toronto, Canada; #L474000, lot 3-GHZ-140-1) were acquired from the Centre Hospitalier Universitaire Vaudois (CHUV, Lausanne, Switzerland) as 20 mg/ml and 10 mg/ml stock solutions in DMSO, respectively. The structure of the drugs used in this work are presented in Fig. S8.

### Lipoprotein isolation

HDLs and LDLs were prepared from human serum (healthy donors, human immunodeficiency virus-, hepatitis B virus- and hepatitis C virus-negative) by sequential density ultracentrifugation (Havel et al., 1955; James and Pometta, 1990).

### Cells and cell culture

Wild-type DLD1 (gift from Prof. Bert Volgenstein at the Core Cell Center Baltimore, USA) cells and HeLa cells [American Type Culture Collection (ATCC) #CCL-2] were maintained in RPMI 1640 (Gibco; ref. 61870-010; lot 1880320) supplemented with 10% fetal bovine serum (FBS; Gibco; ref. 10270-106; lot 42G5062K) at a temperature of 37°C with 5% CO_2_. HEK293T cells (ATCC #CRL-11268), MCF7 cells and mouse embryonic fibroblasts (MEFs; generated from C57BL6 mouse embryos) were maintained in DMEM (Gibco; ref. 61965-059; lot 2205977) supplemented with 10% FBS (Gibco; ref. 10270-106; lot 42G5062K) at a temperature of 37°C with 5% CO_2_. MIN6 clone B1 mouse insulinoma cells (kindly provided by Dr Philippe Halban, University Medical Center, Geneva, Switzerland) were cultured in high-glucose DMEM (Gibco; ref. 61965-026) supplemented with 15% FBS, 1 mM of sodium pyruvate (Gibco; ref. 11360-070) and 70 μM freshly added β-mercaptoethanol (Gibco; ref. 31350-010) at a temperature of 37°C with 5% CO_2_. The HeLa, DLD-1 and HEK293T cell lines were authenticated by Microsynth (Balgach, Switzerland) in December 2021.

### Extracellular and intracellular drug quantitation

DLD-1 cells were plated in six-well plates at a density of 500,000 cells per well and cultured for 24 h in 2 ml of culture medium. The cells were then treated with 20 μM TG or 100 nM staurosporine in 1 ml fresh medium for 2 h. Cells were washed with PBS three times and then incubated in fresh RPMI with 10% FBS in the absence (control) or in the presence of 1 mM HDL. After 2 h, the media were collected in Eppendorf tubes. Cells were washed with PBS and then trypsinized. Cell pellets were kept in Eppendorf tubes. The drug content in the samples was analyzed by high performance liquid chromatography coupled to tandem mass spectrometry (HPLC-MS/MS).

### Drug content analysis by mass spectrometry

To prepare an incubation medium sample, a 300 μl aliquot of the incubation medium sample was mixed with 300 μl MeOH. This mixture was then vortex-mixed, and centrifuged at 16,000 ***g*** for 10 min. The supernatant (500 μl) was transferred into an HPLC glass vial.

Cell samples were prepared by mixing 10^6^ cells with 300 μl MeOH. This cellular suspension was sonicated for 30 min. H_2_O (300 μl) was then added, and the sample centrifuged at 16,000 ***g*** for 10 min to eliminate solid cellular debris. The supernatant (500 μl) was transferred into an HPLC glass vial.

For calibration curves, quantitative analysis of the concentrations was performed using the external standard method. A calibration standard curve was calculated and fitted by quadratic log-log regression of the peak areas. The lower limits of quantification were 50 ng/ml for TG and 1 ng/ml for staurosporine.

### Intracellular drug quantitation by flow cytometry

Cells were plated in six-well plates at a density of 150,000 cells per well in 2 ml RPMI with 10% FBS and cultured for 24 h. Then, the medium was replaced with 1 ml fresh medium containing or not the indicated BODIPY-labeled drugs or naturally fluorescent drugs for 1 h. The cells were then washed once and then left untreated or incubated with the indicated lipoproteins or proteins for various periods of time. Finally, cells were washed once with 1 ml PBS and trypsinized with 120 µl of trypsin-EDTA for ∼2 min and recovered following the addition of 500 µl of RPMI with 10% FBS, centrifuged at ∼200 ***g*** for 3 min and finally resuspended in 500 µl PBS for flow cytometry analysis. The fluorescence of BODIPY-TG, BODIPY-glibenclamide, doxorubicin hydrochloride and Rhodamine 123 fluorescence was measured using the KO525, FITC, PE, and FITC channels of a CytoFlex-S flow cytomter (Beckman), respectively. Data analysis was performed with Kaluza Version 1.3 software.

### siRNA transfection

The first round of siPOOL transfection was performed at the time of cell seeding (80,000 cells/well, 400 μl DMEM, 10% FBS) in 12-well plates. The transfection mix was made as follows: 50 μl Opti-MEM (Thermo Fisher Scientific 11058021) were placed in two different sterile Eppendorf tubes, 1.5 μl siRNA (5 μM stock) were added to one tube and 1.25 μl RNAi-MAX to the other. Then the content of the two tubes were mixed together and incubated at room temperature for 5 min. The mixture was added dropwise in the well of the plates. After 24 h, the medium was replaced with 0.4 ml fresh medium and another siRNA transfection was performed. Cells were analyzed 48 h or 72 h after the first transfection as indicated.

### HEK293T cell number counting

Following siPOOL transfection, HEK293T cells were treated with 20 μM TG for 24 h in the absence or in the presence of 1 mM HDLs. The dead floating cells were removed carefully and the attached cells were collected in 1 ml 10% DMEM and centrifuged at ∼200 ***g*** for 3 min. The cell pellets were then suspended in 0.5–1 ml of DMEM with 10% FBS. A volume of 10 μl was taken and placed on a hemocytometer and cells were counted. The total cell number for each condition were then calculated based on the suspension medium volume.

### Real-time PCR

Cells were treated with unlabeled drugs in a similar manner as described in the previous section except that the pelleted cells after trypsinization and centrifugation were frozen at −80°C until processed for RNA extraction. Total RNA was extracted from cells using High pure RNA isolation kit from Roche (ref. 11828665001, lot 38800800) according to the manufacturer's instructions. Reverse transcription from RNA to cDNA was performed with the Transcriptor Universal cDNA Master kit from Roche (ref. 05893151001, lot 32966400). The semiquantitative real-time PCR was proceeded with the FastStart universal SYBR green master from Roche (ref. 04913914001, lot 11929100) using gene-specific primers. Data were normalized to mRNA levels of *GAPDH* as a housekeeping gene and were analyzed by the 2^−ΔΔCt^ method. Relative expression of genes was expressed as fold change over control. The primers listed in Table S2 were used for the real-time PCR analyses.

### XBP1 splicing

XBP1 splicing assessment was performed according to Zhang and Kaufman (2008) and Fribley et al. (2011). Briefly, RNA was extracted from cells using the High pure RNA isolation kit from Roche (#11828665001). The resulting RNA was quantitated using a Nanodrop 2000c device (Thermofisher). This RNA (10 µl of 20 ng/ml dilutions) was reverse-transcribed using the Transcriptor Universal cDNA Master from Roche (#05893151001). Then, 2 µl of the resulting material was PCR amplified using the following forward and reverse primers. hXBP1 Forward: 5′-CCTTGTAGTTGAGAACCAGG-3′ and hXBP1 Reverse: 5′-GGGGCTTGGTATATATGTGG-3′. The amplified fragments were resolved by a long run (∼10 cm) in 1.8% agarose gel.

### Western blotting

Cells were lysed in lysis buffer (20 mM Tris-HCl pH 7.5, 150 mM NaCl, 1 mM Na_2_EDTA, 1 mM EGTA, 1% Triton, 2.5 mM sodium pyrophosphate, 1 mM β-glycerophosphate, 1 mM Na_3_VO_4_ and 1 µg/ml leupeptin). Protein concentrations in the cell lysates were quantified by Bradford assay. Equal amounts of protein per sample (20–40 µg, depending on the experiment) were loaded in a 10% (12% when CHOP was assessed) polyacrylamide gel; proteins were then transferred to nitrocellulose membranes. Following Ponceau (0.1% Ponceau, 50% acetic acid) staining to confirm proper protein transfer, the membrane was incubated with the indicated primary antibodies using the conditions shown in Table S1, washed for 20 min three times in TBS (25 mM Tris-HCl pH 7.2–7.5, 0.15 M NaCl) with 0.1% Tween 20, incubated with a secondary antibody and washed for 20 min in TBS with 0.1% Tween 20. The blots were finally detected by Odyssey infrared imaging system (LICOR Biosciences, Bad Homburg, Germany).

### SERCA2 knockdown

The mSERCA2-shRNA2.pll3.7 vector (plasmid #748) was used to generate lentiviruses encoding an shRNA against SERCA2 (the sequence targeted by this shRNA is conserved between humans and mice). It was constructed by subcloning annealed oligonucleotides #912 and #913 into the pLentiLox3.7 lentiviral vector (plasmid #627; Addgene #11795) opened with HpaI and XhoI. The sequences of the oligonucleotides were:

#912 (sense): 5′-T**GCAACTGTCTATTTCTGCT**TTCAAGAGA*AGCAGAAATAGACAGTTGC**TTTTTT**C*-3′ (regular font, nucleotide used to complete the U6 promoter; bold, nucleotides 3735–3753 of mouse SERCA2 NM_027838; underlined, shRNA loop; italics, nucleotides 3753–3735 of mouse SERCA2 NM_027838; bold italics, linker; underlined italics, N1 nucleotide of the XhoI site).

#913 (anti-sense): 5′-TCGAG**AAAAAA**GCAACTGTCTATTTCTGCT*TTCTCTTGAA**AGCAGAAATAGACAGTTGC**A*-3′ (regular font, N1–N5 nucleotides of the XhoI site; bold, linker; underlined, nucleotides 3735–3753 of mouse SERCA2 NM_027838; italics, shRNA loop; bold italics, nucleotides 3753–3735 of mouse SERCA2 NM_027838; underlined italics, nucleotide used to complete the U6 promoter).

Recombinant lentiviruses were produced in HEK293T cells (Zheng et al., 2019a). Briefly, cells were co-transfected using the calcium phosphate DNA precipitation method with the lentiviral mSERCA2-shRNA2.pll3.7 vector (plasmid #748), the envelope protein-coding plasmid (pMD2.G, plasmid #554) and the packaging construct (pSPAX2, plasmid #842). After a 6 h transfection period, medium was replaced with fresh medium. After 48 h, the virus-containing medium was harvested and kept at −80°C.

DLD-1 cells (20,000 cells per well) were seeded in six-well plates. The following morning, cells were infected with lentiviruses encoding the shRNA directed against SERCA2. The infection rate was quantitated by monitoring GFP fluorescence after three days. The minimal volume of viruses leading to GFP expression in 100% of the cells (assessed by flow cytometry) was used in subsequent experiments.

### Propidium iodide cell viability assay in DLD-1 cells

After the indicated treatment, cells (including the floating cells in the medium) were collected and then incubated with 8 μg/ml propidium iodide (PI) in PBS for 5 min at room temperature. PI incorporation into cells was then analyzed by flow cytometry.

### Pycnosis assessment in Min6 cells

After the indicated treatment, Min6 cells were fixed with 4% paraformaldehyde (PFA) and incubated for 5 min with 10 μg/ml Hoechst 33342. Pycnotic cells was then determined visually using fluorescence microscopy. At least 700 cells were counted for each condition.

### CRISPR/Cas9-mediated gene disruption

Plasmids hABCB1(sgRNA-1).ltiCRISPRv2 (#1084) and hABCG1 (sgRNA-1).ltiCRISPRv2 (#1085) targeting exon 5 of ABCB1 and exon 2 of ABCG1, respectively, were generated according to a previously described protocol (Ran et al., 2013) using the following oligonucleotides: oABCB1_sgRNA_fwd (#1571) 5′-CACCGTGACAAGTTGTATATGGTGG-3′, ABCB1_sgRNA_rev (#1572) 5′-AAACCCACCATATACAACTTGTCAC-3′, ABCG1_sgRNA_fwd (#1573) 5′-CACCGACTGAGACGGACCTGCTGAA-3′, ABCG1_sgRNA_rev (#1574) 5′-AAACTTCAGCAGGTCCGTCTCAGTC-3′. The lentiviral LentiCRISPRv2 plasmid that was used to generate the ABC transporter-specific sgRNA-encoding plasmids was Addgene plasmid #52961 (deposited by Feng Zhang; Sanjana et al., 2014).

Disruption of the ABCB1 and ABCG1 genes using the CRISPR/Cas9 technology was performed in DLD-1 cells as described previously (Conde-Rubio et al., 2021). ABCB1 knockout (KO) cells were first generated and then used to knockout ABCG1. Knocking out ABCB1 was achieved using transient transfection of plasmid hABCB1(sgRNA-1).ltiCRISPRv2 (#1084) in DLD-1. For the transfection, Lipofectamine 2000 (Invitrogen, ref. 11668-019, lot 2185347) was used according to manufacturer's instructions. Briefly, 2×10^5^ cells were plated in six-well plates (Corning, ref. 3516) containing 2 ml RPMI 1640 (Gibco, ref. 61870-010, lot 2340185). The following day, each well of cells was transfected by the addition of 2 µg plasmid hABCB1(sgRNA-1).ltiCRISPRv2 (#1084) and 2 µl Lipofectamine 2000 mixed with 200 µl Opti-MEM (Thermo Fisher Scientific, ref. 11058021, lot 2177581). After 20 min of incubation at room temperature, the mixture was added dropwise in the wells containing the cells. Cells that were not transfected were eliminated by the addition of 3 µg/ml of puromycin (Thermo Fisher Scientific, ref. A1113802) at 24 h post-transfection, for a duration of 5 days. Clone isolation was performed by limiting dilution in 96-well plates (Corning, ref. 3599). Five ABCB1 knockout clones were obtained using this procedure. The presence or the absence of ABCB1 was assessed by western blotting. Clone 2 was found to lack ABCB1 (Fig. S7A) and was then used for knocking out ABCG1 by lentivirus infection. Lentivirus was produced in HEK293T cells, which were plated in 10 cm dishes at 10^6^ cells/plate in 10 ml RPMI 1640 (Gibco, ref. 61870-010), and left to adhere overnight. The cells were transfected using the calcium phosphate method with 7.5 µg psPAX2 (#842; Addgene #12260), 2.5 µg pMD2.G (#554; Addgene #12259), and 10 µg hABCG1(sgRNA-1).ltiCRISPRv2 (#1085) plasmids. Briefly, chloroquine (Sigma-Aldrich, ref. C6628) was added to the medium to a final concentration of 25 µM. In a sterile tube, the DNA and sterile water were mixed to a final volume of 450 µl. After the addition of 50 µl of 2.5 M CaCl_2_ solution, the samples were mixed and incubated for 20 min at room temperature. Then, 500 µl of a 2× HEPES solution (NaCl 280 mM, KCl 10 mM, Na_2_HPO_4_ 1.5 mM, D-glucose 12 mM, HEPES 50 mM) was added and the tube contents were mixed. At 1 min after the HEPES buffer was added, the contents of the tube were added dropwise to the cells. After 16 h, the medium was changed and the cells were grown for 48 h more. Next, the medium was collected and centrifuged at 3000 ***g*** for 5 min to remove non-viable cells. The supernatant containing the viral particles was directly used to infect the ABCB1 knockout clone 2 DLD-1 cells. Infection was performed via titration of virus added to infected cells in the presence of puromycin selection at 3 µg/ml. After 5 days of selection in the presence of puromycin, clone isolation was performed by limiting dilution in 96-well plates (Corning, ref. 3599). Fourteen clones were obtained using this procedure. The presence or the absence of ABCG1 was assessed by analyzing the sequences in the vicinity of the region targeted by the CRISPR/Cas9 system using TA cloning following the procedure outlined in the ‘TA cloning’ section below. One validated ABCB1/ABCG1 double knock-out clone (clone BG1; Fig. S7B) was subsequently used in functional assays.

### TA cloning

DLD-1 clones (250,000 cells) were cultured in wells of six-well plates in RPMI 10% FBS in 5% CO_2_, 37°C incubator for 16 h. The cells were then scraped and resuspended in 100 µl of DNA extraction solution A (NaOH 25 mM, EDTA 0.2 mM) and incubated 30 min at 95°C in a thermoblock set to shake at 600 rpm. The samples were then placed on ice for 5 min. A volume of 100 µl of DNA extraction solution B (40 mM Tris-HCl pH 5.0) was then added, the samples were vortexed 10 s and centrifuged at 16,000 ***g*** for 10 min at 20°C. The DNA concentration of the supernatants was measured using a Nanodrop 2000c apparatus (Thermo Scientific).

ABCG1 exon 2 of the DNA samples (100 ng) was PCR amplified (501 bp fragment) using forward primer hABCG1_F (#1615; 5′-AGGTGGGCACATTTCTCCTG-3′; nucleotides 7324–7343 of human ABCG1 gene; NCBI entry AB038161.1) and reverse primer hABCG1_R (#1616; 5′-TCAGCCTCTCAACCTCCAGA-3′; nucleotides 7824–7805 of human *ABCG1* gene; NCBI entry AB038161.1). The PCR was performed in 50 µl made of the FastStart PCR buffer (2 mM MgCl_2_ final) from Roche (#12161567001, lot 17102900), containing 0.2 mM dNTPs (Promega #U51B, lot 0000234024), 0.008 U/µl Taq DNA polymerase (Roche #11647687001, lot 12508225), and 1 µM of each primer. PCR amplification was done in a Biometra T1 plus thermocycler as follows: 4 min at 95°C, then 38 cycles of 30 s at 95°C, 30 s at 55°C, 45 s at 72°C, then a last incubation at 72°C for 4 min and finally the samples kept at 4°C. Correct amplification was checked by running 5 µl of the PCR reactions on a 1% agarose gel. The PCR fragments were then purified in a final 20 µl water volume using the Wizard SV Gel and PCR Clean-Up system from Promega (#A9282, lot 0000262975) as per the manufacturer's instructions and quantitated using the Nanodrop 2000c device.

A total of 6 ng of the purified PCR fragments was then ligated to 50 ng of opened pCR2.1 vectors that accept PCR fragments with A overhangs (Invitrogen, #46-0572; lot 2266680). The reaction was performed in 10 µl containing 2 µl 5X T4 DNA ligase reaction buffer (Invitrogen, #PN 100017419; lot 2209191), and 1 µl of ExpressLink T4 DNA ligase (5 U/µl; #PN 100017418; lot 2209192) and incubated 20 min at room temperature, 10 min at 16°C and finally 18 h at 4°C. Half the reaction (5 µl) was then mixed with 50 µl of Subcloning Efficiency DH5α competent bacteria and incubated on ice for 30 min, heat shocked at 42°C for 30 s, placed back on ice for 2 min, and transferred to round-bottom 15 ml tubes containing 250 µl of LB (10 g/l tryptone, 10 g/l sodium chloride, 5 g/l yeast extract; Conda Laboratories, #1551.0, batch number 804091). The samples were next incubated 1 h at 37°C with 220 rpm shaking and then spread on lysogeny broth (LB), 50 µg/ml kanamycin plates and incubated at 37°C for 24 h. Colonies were then picked with yellow tips and transferred to 15 ml tubes containing 3 ml of LB, 50 µg/ml kanamycin that were incubated at 37°C for a 18.5 h period with 220 rpm shaking. Plasmids were purified using the Qiagen QIAprep Spin Miniprep Kit (#27104) according to the manufacturer's instructions (including the PB optional wash) and quantitated using the Nanodrop 2000c device. The plasmids (300 ng) were sequenced by Fasteris, Life science Genesupport SA (Plan-les Ouates, Switzerland) using the T7 primer.

### Data presentation and statistics

Data from independent experiments are depicted with symbols of different motifs and shading. Comparisons between multiple groups were performed using two-way ANOVA or one-way ANOVA followed by Dunnett's multiple comparisons test using GraphPad Prism. Unless otherwise mentioned, comparisons between two groups were performed using a two-tailed paired Student's *t*-test in GraphPad Prism.

## Supplementary Material

Supplementary information

Reviewer comments
