# Supplementary material for: HDLs extract lipophilic drugs from cells
Source: J Cell Sci. 2022 Jan 31;135(5):jcs258644. doi: 10.1242/jcs.258644 (PMC8919334; doi:10.1242/jcs.258644)
Supplement: Supplementary information [file joces-135-258644-s1.pdf]

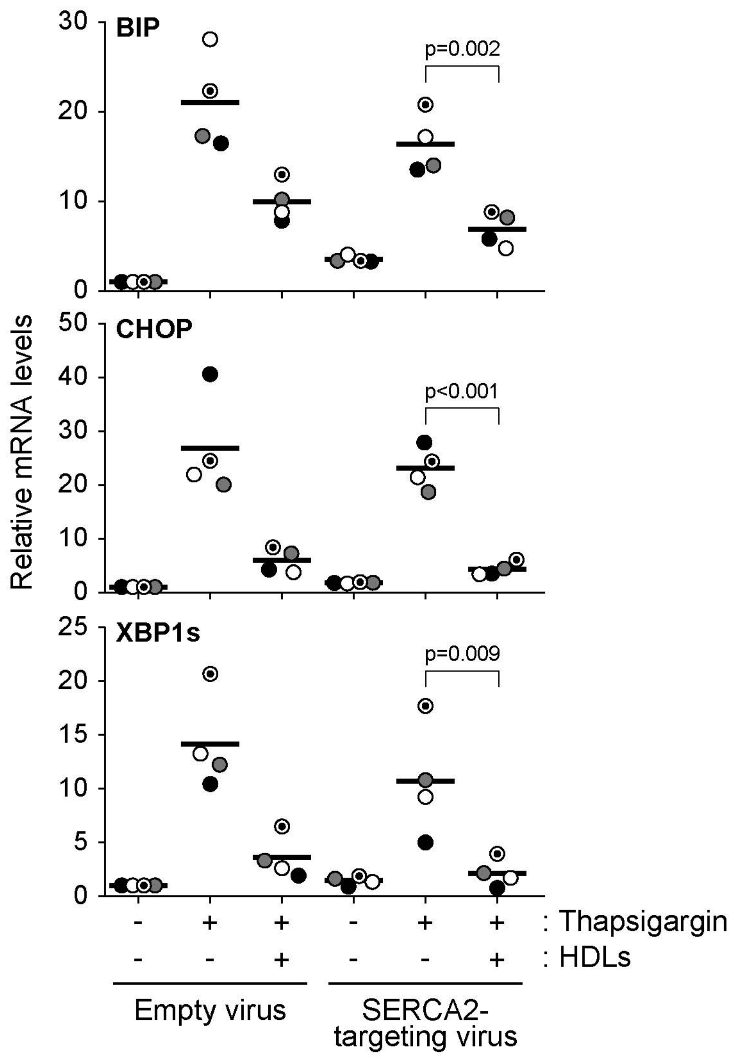

Figure S1

Fig. S1. The effect of HDLs on TG-induced ER stress in SERCA2 knockdown DLD-1 cells.

DLD-1 cells that were infected with a control virus or a lentivirus encoding for an shRNA directed against SERCA2 (see Figure 3B) were seeded in 6-well plates (200000 per well). Twenty four hours later, cells were treated or not with TG (15 μM) in the presence or in the absence of 1 mM HDL for an additional 24 hour period. The mRNA expression levels of the indicated ER stress markers were measured by RT-PCR.

Journal of Cell Science • Supplementary information

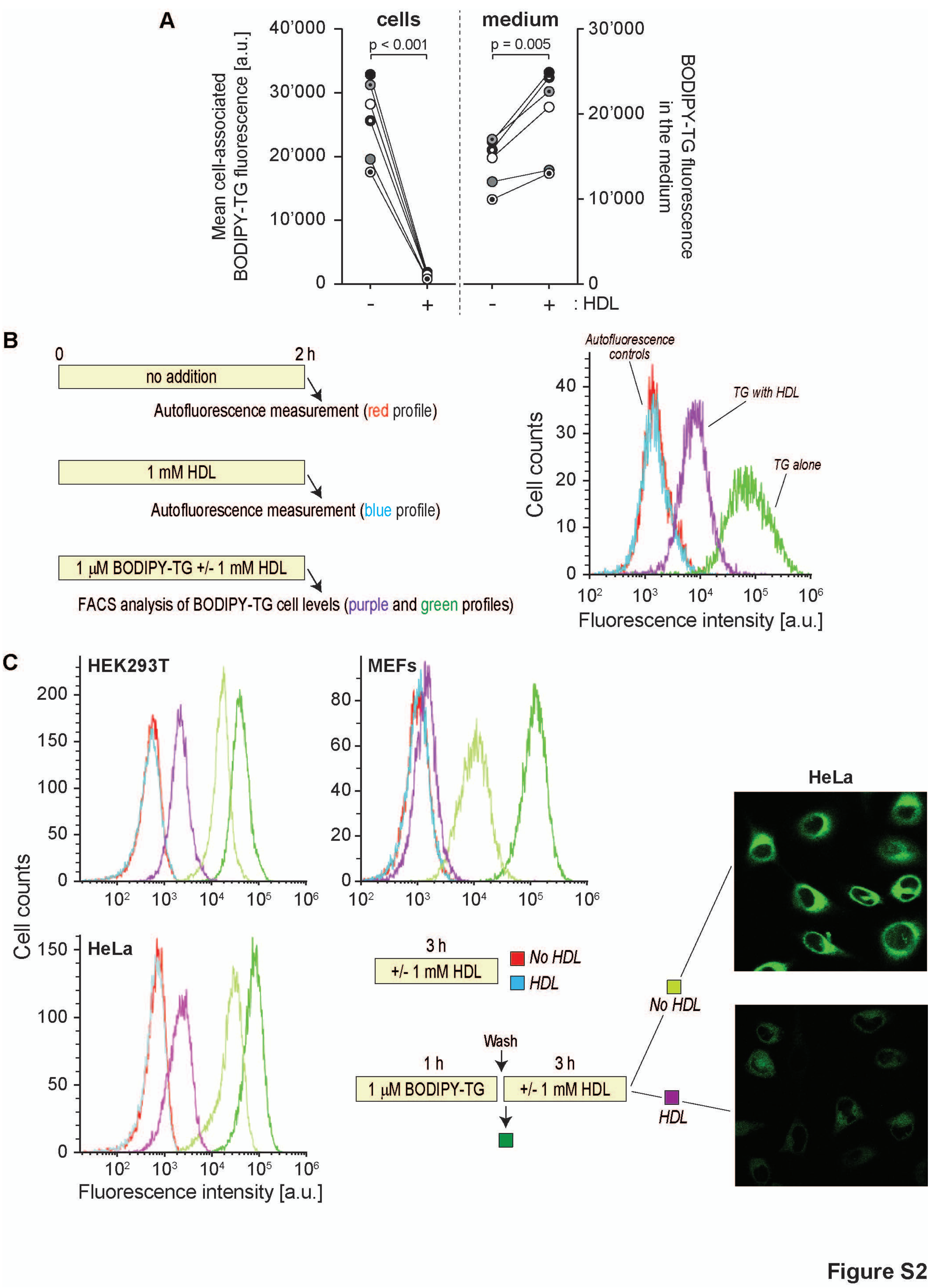

Figure S2

Fig. S2. HDL-mediated BODIPY-TG extraction

A. Hela cells (150 000 cells) were plated in 6-well plates. Twenty four hours later, they were treated with 1 µM BODIPY-TG for 1 hour, washed once with PBS, and then incubated in the absence or in the presence of 1 mM HDL for another 3 hours. The cell-associated BODIPY-TG levels were assessed by flow cytometry (left hand side graph labelled “cells”) and those in the medium measured using a Cytation 3 cell imaging multi-mode reader (excitation wavelength: 485 nm; emission wavelength: 538 nm) (right hand side graph labelled “medium”). The results are derived from 6 independent experiments (each labelled with a unique symbol). **B**. Min6 cells (300 000 cells per well) were treated with 1 µM BODIPY-TG in the presence or in the absence of 1 mM HDL for 2 hours. The cells were then collected and fluorescence intensity was monitored by flow cytometry. The autofluorescence profiles of untreated and HDL-incubated cells are also shown. **C**. Cells were seeded in 6 well plates (150000, 80000, and 200000 per well for HEK293T, MEFs, and HeLa cells, respectively) one day before being treated as described in the scheme. Cells were then analyzed by flow cytometry. The images on the right hand side correspond to confocal imaging of 120000 HeLa cells seeded the previous day in 35 mm glass bottom dishes and treated as shown in the scheme. The recording confocal settings between the two images were identical.

Journal of Cell Science • Supplementary information

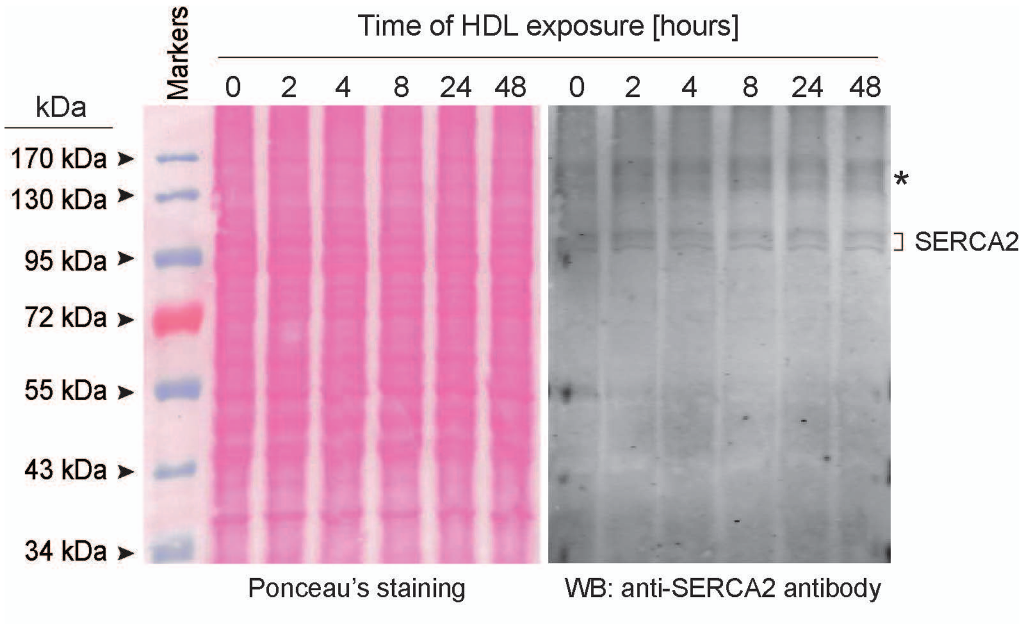

Figure S3

Fig. S3. HDLs do not modulate SERCA levels.

DLD1 were seeded in 6-well plates (200000 cells per well except for the “48 hours” condition where 100 000 cells were seeded). Twenty four hours later, they were exposed to 1 mM HDLs for the indicated time periods (time 0 corresponds to no HDL exposure). SERCA2 expression was assessed by western blotting and loading evaluated via Ponceau staining. Asterisk indicates non-specific bands.

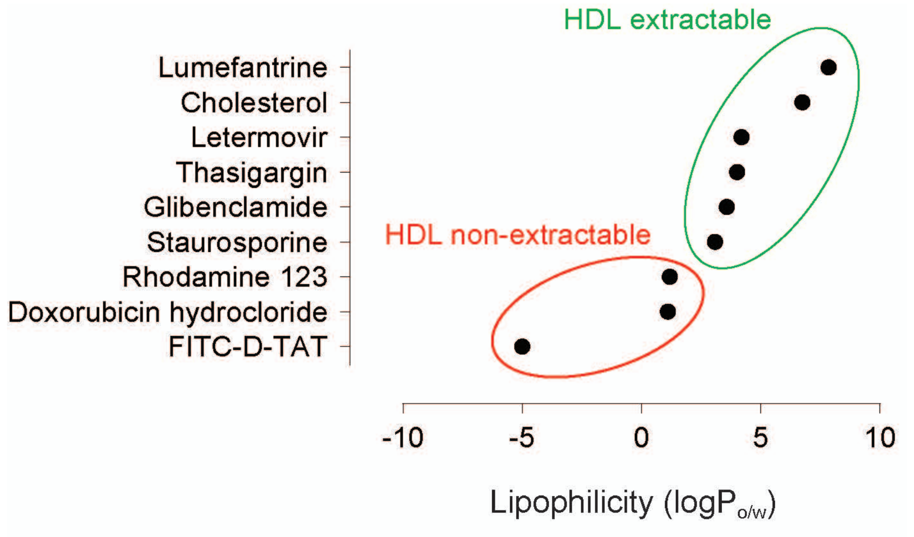

Figure S4

Fig. S4. Correlation between HDL-mediated efflux efficiency and drug lipophilicity. The n-octanol/water partition coefficient (log $P_{o/w}$) of the indicated compounds was obtained through the SwissADME website (http://www.swissadme.ch) (Daina et al., 2017).

Journal of Cell Science • Supplementary information

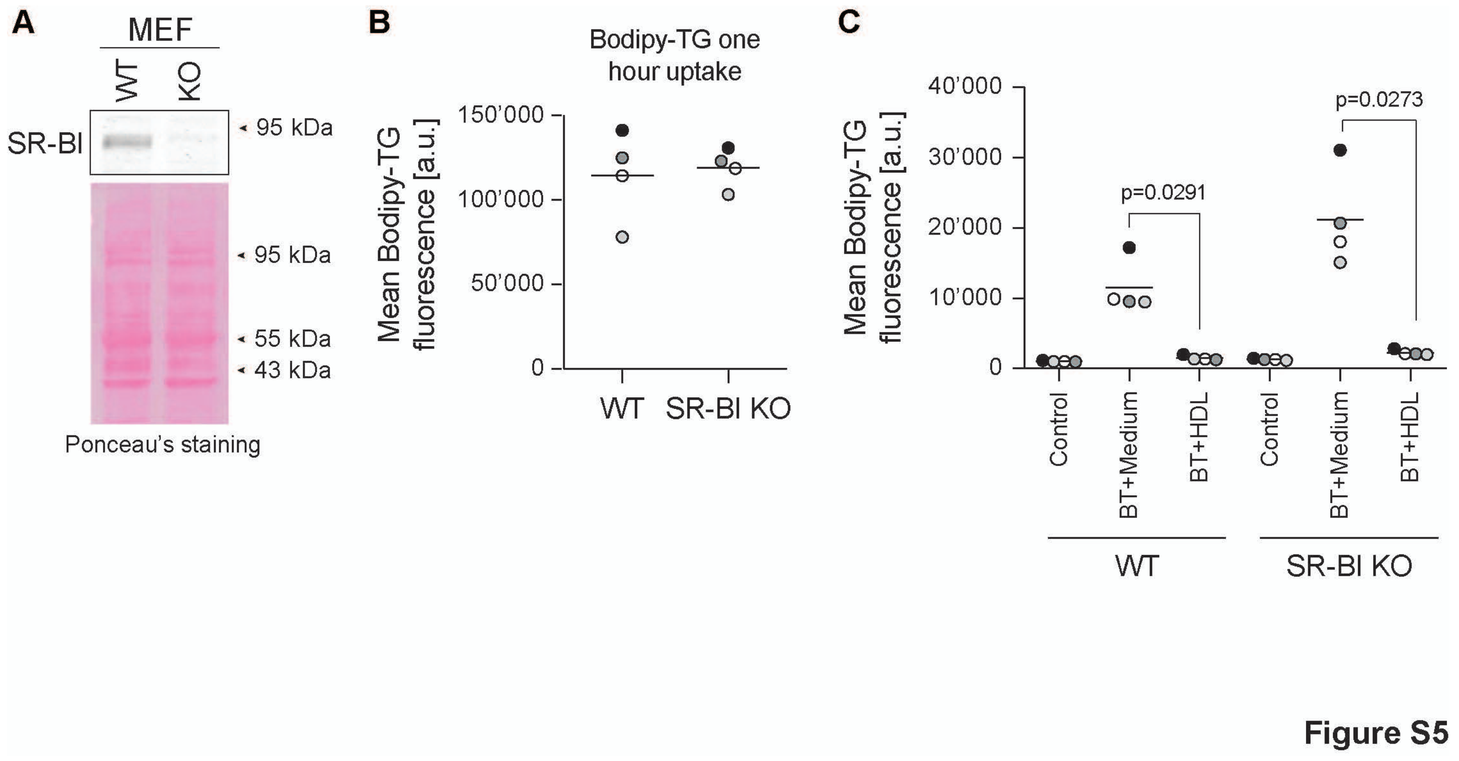

Fig. S5. Role of SR-BI on BODIPY-TG efflux.

A. SR-BI expression in wild-type (WT) and SR-BI knockout (KO) MEF cells. **B**. BODIPY-TG uptake in WT and SR-BI KO MEF cells. **C**. BODIPY-TG efflux in WT and SR-BI KO MEF cells.

Journal of Cell Science • Supplementary information

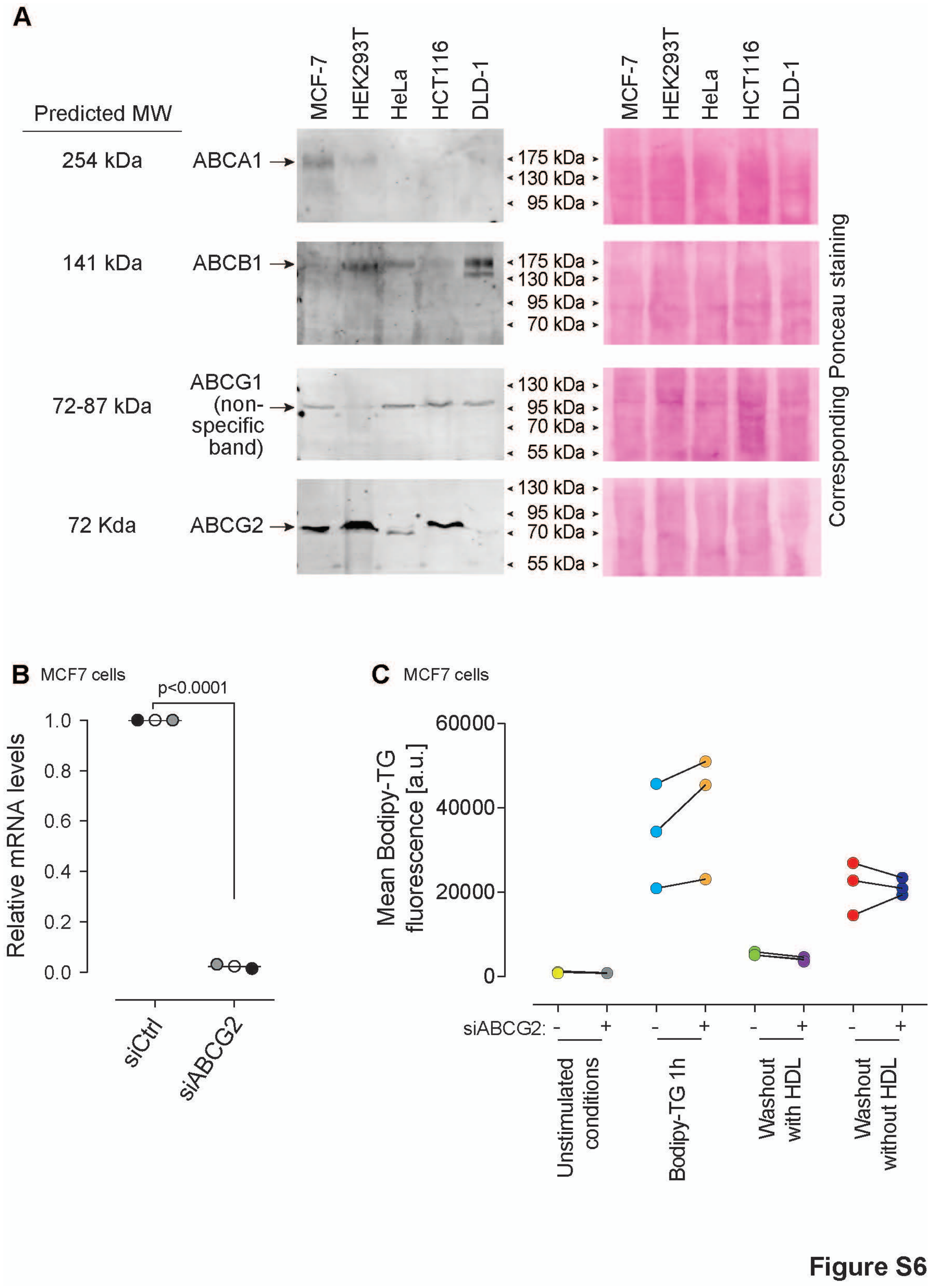

Fig. S6. Role of ABCG2 on BODIPY-TG efflux in HEK293T cells.

A. ABC transporter expression in the indicated cell lines was assessed by Western blotting. The indicated molecular weight (MW) were obtained from the Uniprot website. Note that the ABCG1 gene can give rise to 8 different isoforms. This explains the range of MW for this transporter. As reported in Figure S7, the anti-ABCG1 antibody was found to be not specific for this transporter. The indicated band corresponds therefore to a non-specific interaction. **B-C**. MCF7 cells (80000 per well) were plated in a 12-well plate. Then cells were transfected with a pool of siRNA directed at ABCG2 and analyzed 72 hours post-transfection. Knockdown efficiency was assessed at the mRNA level by RT-PCR (panel B). Alternatively, cells were treated as indicated in the scheme shown in Figure 8C. Cell-associated drug fluorescence was measured by flow cytometry. The graphs present the data derived from 3 independent experiments (panel C).

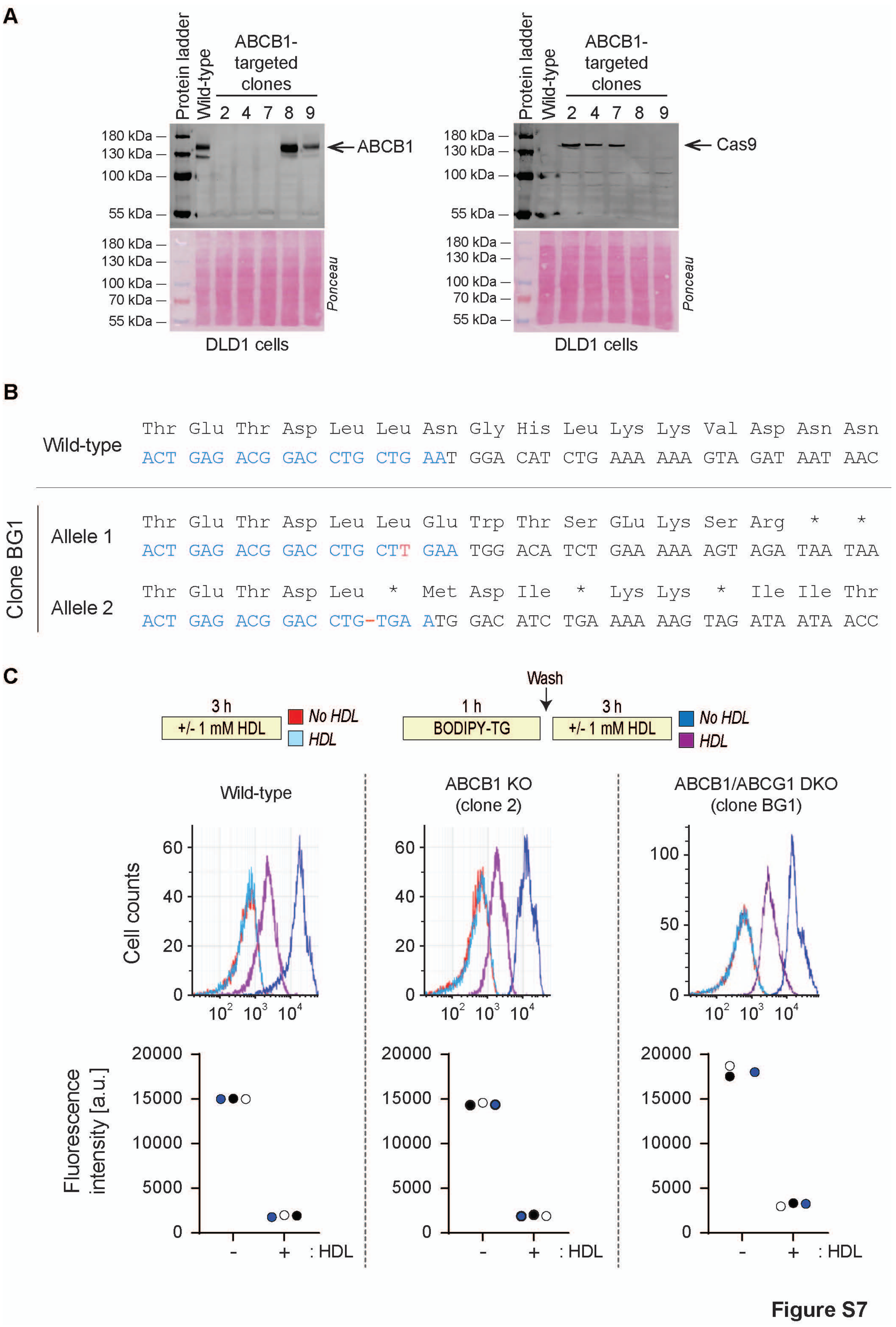

Fig. S7. Role of ABC transporters on BODIPY-TG efflux in DLD1 cells.

A. ABCB1 gene invalidation in DLD1 cells. The presence of ABCB1 and Cas9 in wild-type and CRISPR/Cas9 ABCB1-targeted clones was assessed by Western blotting. Loading evenness was evaluated by Ponceau staining. Note that the clones in which ABCB1 expression was abrogated were those that still expressed the Cas9 protein. **B**. CRISPR/Cas9-mediated ABCG1 gene disruption in the ABCB1 knock-out clone n°2, generating clone BG1. The sequence in blue corresponds to the sgRNA binding site in ABCG1 exon 2. The T nucleobase written in red is an insertion. The amino acids encoded by the codons are shown above the DNA sequences. The asterisks denote stop codons. The red dash indicates deletion of a cytosine nucleobase. **C**. The indicated cells (120000 per well) were plated in a 6-well plates. They were then treated as indicated in the scheme above the graphs. BODIPY-TG cell-associated fluorescence was assessed by flow cytometry (one representative experiment shown). The quantitation (mean of the cytometry distribution profiles) of 3 independent experiments (labelled with different symbols) are depicted below the flow cytometry profiles.

Journal of Cell Science • Supplementary information

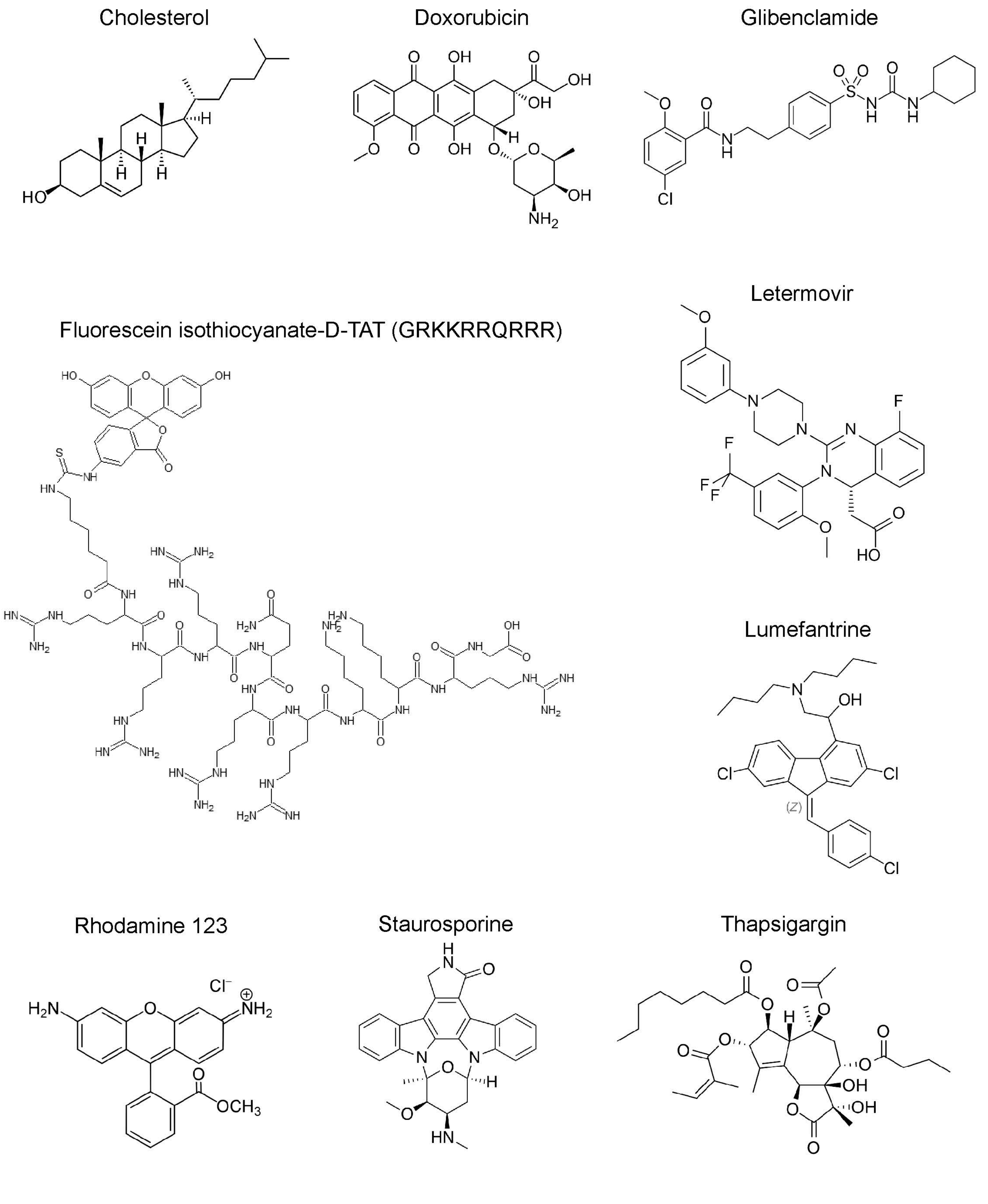

Figure S8

Fig. S8. Structures of the drugs used in this work.

Journal of Cell Science • Supplementary information

Table S1. Antibodies used in this work

Antigen or antibody type	Dilution	Incubation buffer	Incubation temperature	Incubation time	Vendor	Reference	Lot number
BIP	1:1000	TBS-tween 0.1%, 5% milk	4°C	Overnight	Cell Signaling	3183	6
CHOP	1:500	TBS-tween 0.1%, 5% milk	4°C	48 hours	Cell Signaling	2895	13
SERCA1	1:1000	TBS-tween 0.1%, 5% milk	4°C	Overnight	Abcam	Ab2819	GR3278625-1
SERCA2	1:1000	TBS-tween 0.1%, 5% milk	4°C	Overnight	Cell Signaling	4388	2
SERCA3	1:1000	TBS-tween 0.1%, 5% milk	4°C	Overnight	Abnova	H00000489-M01	I951-2H3
ABCA1	1:1000	TBS-tween 0.1%, 5% milk	4°C	Overnight	Abcam	Ab66217	GR113106-1
ABCB1	1:500	TBS-tween 0.1%, 5% milk	4°C	Overnight	Abcam	Ab170904	GR32192362
ABCG1	1:1000	TBS-tween 0.1%, 5% milk	4°C	Overnight	Abcam	Ab52617	GR3255111-4
ABCG2	1:1000	TBS-tween 0.1%, 5% milk	4°C	Overnight	Abcam	Ab207732	GR3232144-1
SR-BI	1:500	TBS-tween 0.1%, 5% milk	4°C	Overnight	Novus Biologicals	NB400-104SS	O
Alexa Fluor 680 goat anti-rabbit antibody	1:5000	TBS-tween 0.1%, 5% milk	Room temperature	1 hour	ThermoFisher	A21109	1816534
Alexa Fluor plus 800 goat anti-rabbit antibody	1:5000	TBS-tween 0.1%, 5% milk	Room temperature	1 hour	ThermoFisher	A32735	RJ243469
Alexa Fluor 680 goat anti-mouse antibody	1:5000	TBS-tween 0.1%, 5% milk	Room temperature	1 hour	ThermoFisher	A21057	1752100
Alexa Fluor 680 goat anti-rat antibody	1:5000	TBS-tween 0.1%, 5% milk	Room temperature	1 hour	ThermoFisher	A21096	1718849

Journal of Cell Science • Supplementary information

Table S2. Primers used for RT-PCR-based detection of the indicated proteins (h, human).

Target	Directio n	Number	Sequence	Description	NCBI entry
h-SERCA2	Forward	1612	ATG GGG CTC CAA CGA GTT AC	nucleotides 648-667 of human SERCA2, variant a; identical sequence in human variant b	NM_001681.4
h-SERCA2	Reverse	1613	TTT CCT GCC ATA CAC CCA CAA	nucleotides 851-871 of human SERCA2, variant a	NM_001681.4
h-BIP	Forward	1617	GAA AGA AGG TTA CCC ATG CAG T	nucleotides 702-723 of human BIP	NM_005347.5
h-BIP	Reverse	1618	CAG GCC ATA AGC AAT AGC AGC	nucleotides 830-850 of human BIP	NM_005347.5
h-XBP1s	Forward	1619	TGC TGA GTC CGC AGC AGG TG	nucleotides 528-547 of human XBP1,variant 2	NM_001079539.2
h-XBP1s	Reverse	1620	GCT GGC AGG CTC TGG GGA AG	nucleotides 677-696 of human XBP1,variant 2	NM_001079539.2
h-CHOP	Forward	1621	GGA AAC AGA GTG GTC ATT CCC	nucleotides 491-511 of human CHOP, variant 5; identical sequence in variants 1-4 and 6	NM_004083.6
h-CHOP	Reverse	1622	CTG CTT GAG CCG TTC ATT CTC	nucleotides 586-606 of human CHOP, variant 5; identical sequence in variants 1-4 and 6	NM_004083.6
h-ABCB1	Forward	1602	TTG CTG CTT ACA TTC AGG TTT CA	nucleotides 897-919 of human ABCB1,variant 1; identical sequence in variants 2-4	NM_001348945.2
h-ABCB1	Reverse	1603	AGC CTA TCT CCT GTC GCA TTA	nucleotides 981-1001 of human ABCB1,variant 1; identical sequence in variants 2-4	NM_001348945.2
h-GAPDH	Forward	1578	CTG ACT TCA ACA GCG ACA CC	nucleotides 873-892 of human GAPDH,variant 7; identical sequence in variants 1-4	NM_001357943.2
h-GAPDH	Reverse	1579	TGC TGT AGC CAA ATT CGT TG	nucleotides 967-986 of human GAPDH, variant 7; identical sequence in variants 1-4	NM_001357943.2
h-ABCG2	Forward	1606	CAG GTG GAG GCA AAT CTT CGT	nucleotides 350-370 of human ABCG2, variant 7; identical sequence in variants 1-6	NM_001348989.2
h-ABCG2	Reverse	1607	ACC CTG TTA ATC CGT TCG TTT T	nucleotides 575-596 of human ABCG2, variant 7; identical sequence in variants 1-6	NM_001348989.2

Journal of Cell Science • Supplementary information
