# Supplementary material for: HDLs extract lipophilic drugs from cells
Source: J Cell Sci. 2022 Jan 31;135(5):jcs258644. doi: 10.1242/jcs.258644 (PMC8919334; doi:10.1242/jcs.258644)
Supplement: Reviewer comments [file joces-135-258644_review_history.pdf]

HDLs extract lipophilic drugs from cells

Adi Zheng, Gilles Dubuis, Maria Georgieva, Carla Susana Mendes Ferreira, Marc Serulla, Maria del Carmen Conde Rubio, Evgeniya Trofimenko, Thomas Mercier, Laurent Decosterd and Christian Widmann

DOI: 10.1242/jcs.258644

Editor: James Olzmann

Review timeline

Submission to Review Commons:	10 December 2020
Submission to Journal of Cell Science:	12 March 2021
Editorial decision:	16 March 2021
First revision received:	1 December 2021
Accepted:	14 December 2021

Reviewer 1

Evidence, reproducibility and clarity

It was my pleasure to evaluate the work submitted to Review Commons. I have reviewed the work and my comments are as follows:
This manuscript entitled "HDLs extract lipophilic drugs from cells" by Zheng and colleagues describes a new mechanistic picture of how HDLs protect cells against death. The authors meticulously describe a novel ability of HDLs to extract hydrophobic xenobiotics from cells akin to their cholesterol-extracting function. I would like to thank the authors for a pleasurable read and their well-defined experimental design. This manuscript is of great value and significance to the fields of clinical chemistry and pharmacology. I therefore do think this manuscript merits publication after tending to these major and minor comments.

Major points:

- A logical question comes up and I do not think the authors addressed, in a human body what happens to the extracted drugs after loading on HDLs? This requires some mentioning in the discussion.
- Is the effect specific to the fully mature HDL molecule or do apo-lipoproteins that compose HDLs have similar effects?
- What are non-SERCA-mediated effects of TG?
- Why don't HDLs protect cells from low dose TG despite its removal?
- Line 144. No information on the siRNA was given (refer to the materials section to guide the reader).

Minor comments:
- There needs to be an abbreviation section. Make sure that you only abbreviate the terms that are used more than once in the text.
- Lines 104, 277, 283 and anywhere else: use TG instead of thapsigargin.
- Line 262: you don't have to redefine SERCA.
- I suggest adding structures of the used drugs.
- I suggest using a table for the RT-PCR primers.

© 2021. Published by The Company of Biologists under the terms of the Creative Commons Attribution License (https://creativecommons.org/licenses/by/4.0/).

Protein Direction Number Sequence Description NCBI entry
h-SERCA2 Fwd #1612 5'ATG GGG CTC CAA CGA GTT AC nucleotides 648-667 of human SERCA2, variant a NM_001681.4
- Line 93: DMEM (Gibco; ref 61965-059;) the lot number is missing.
- Line 102: 500'000 (and all other thousand numbers) the apostrophe's place is strange.
- Line 381: cholesterol carriers.

Significance

This manuscript is of great value and significance to the fields of clinical chemistry and pharmacology.

Referees cross-commenting

I agree with the experiments suggested by reviewer #2

Reviewer 2

Evidence, reproducibility and clarity

In this manuscript, Christian Widmann and colleagues describe how HDLs can protect cells by promoting the extraction of lipophilic drugs such as thapsigargin (TG). The authors observe that HDLs do not affect the ability of TG to inhibit SERCA but instead decrease lipophilic drug content inside cells and therefore protect cells against their lethal effects. Using some compounds (probably not enough to conclude), the authors claim that HDLs can promote the exclusion of lipophilic drugs while hydrophilic drugs or compounds like doxorubicin hydrochloride, an anticancer drug, or Rhodamine 123, were not extracted from cells. Finally using small interfering RNA, the authors reveal that ABCB1 mediates some of the drug effluxes to HDLs.
This study is sound and well-written. Although of interest from a therapeutic standpoint, this manuscript should address some questions to strengthen these data.

Major concerns
1. Figure 2, The authors should perform western blot to evaluate the protein expression levels (not only mRNA levels by Q-PCR)

2. Could the authors evaluate whether HDL treatment reduces the amount of SERCA (mRNA/protein) in their cells? The loss of SERCA could explain the reduced accumulation of the BODIPY-TG in the cell?

3. To generalize their observation, It would have been interesting to test more lipophilic/hydrophilic drugs to quantitatively validate that HDLs are selective of lipophilic drugs.

4. The ABC transporter part in this manuscript has to be improved with the down-regulation of extinction of ABCA1 and ABCG1 to determine in a comprehensive manner the effect of these transporters in the pro-survival role of HDL.

Minor point:
1. ABCB1 blot in figure 7B is not convincing and should be improved.

© 2021. Published by The Company of Biologists under the terms of the Creative Commons Attribution License (https://creativecommons.org/licenses/by/4.0/).

Significance

This study can interest a large scientific audience.
Some additional experiments have to be performed to render more convincing some part of this study.

Author response to reviewers' comments

We thank both reviewers for their insightful comments and suggestions. We propose to address these as described below.

Reviewer 1

Major points:

Point 1
Q. A logical question comes up and I do not think the authors addressed, in a human body what happens to the extracted drugs after loading on HDLs? This requires some mentioning in the discussion.
A. This is indeed a good question. We have now added in the discussion what may happen to the HDL-extracted drugs in a whole organism. It reads as follows:
The likely fate of HDL-extracted drugs in humans is that they are carried to the liver by HDLs. Scavenger receptors such as SR-BI expressed by hepatocytes can then bind HDLs carrying the extracted drugs allowing the drugs to be taken up by the cells. In hepatocytes, the drugs may be inactivated and excreted in the bile (https://doi.org/10.1016/j.cld.2016.08.001, https://doi.org/10.1161/CIRCRESAHA.119.312617).

Point 2
Q. Is the effect specific to the fully mature HDL molecule or do apo-lipoproteins that compose HDLs have similar effects?
A. This is an interesting question. Apo-AI is the characteristic and most abundant apolipoprotein found in HDLs. It is however not trivial to compare the activities of ApoAI and HDLs because of the difficulty of producing large amounts of ApoAI. In the present paper, the lowest concentration of HDLs that induces drug efflux is 0.125 mM. As there are about 3 molecules of Apo-AI per HDL molecule, we should use 0.375 (3 x 0.125) mM Apo-AI to see if the Apo-AI content of these HDLs can mediate or mimic the drug efflux capacity of the lipoproteins. About 100 mg of recombinant Apo-AI would be required to make 10 ml of a ~0.3 mM Apo-AI cell culture solution. This is an enormous task requiring substantial time and money investment. We are therefore not in a position to perform this experiment that would be of interest but which is not central for supporting the main message of our manuscript.

Point 3
Q. What are non-SERCA-mediated effects of TG?
A. The SERCA-independent toxic effects of TG have been shown to be a consequence of mitochondrial dysfunction resulting from the ability of TG to induce mitochondrial permeability transition (DOI: 10.1046/j.1432-1327.1999.00724.x). This is now mentioned in the discussion.

Point 4
Q. Why don't HDLs protect cells from low dose TG despite its removal?
A. Our data indicate indeed that HDLs do not affect the ability of TG to inhibit SERCA and the low ER stress response that ensues. This can be explained by the fact that very low concentrations of TG inhibit SERCA in an irreversible manner (Ki values of 0.2, 1.3, and 12 nM for SERCA1b, SERCA2b, and SERCA3a, respectively) (DOI:https://doi.org/10.1074/jbc.M510978200). Hence, even though HDLs can remove a substantial amount of TG from cells, the concentration of TG that remains in cells is presumably still sufficient to fully inhibits the SERCA pumps. This explanation is now included in the discussion.

© 2021. Published by The Company of Biologists under the terms of the Creative Commons Attribution License (https://creativecommons.org/licenses/by/4.0/).

Point 5
Q. Line 144. No information on the siRNA was given (refer to the materials section to guide the reader).
A. The siPOOLs we have used correspond, for each targeted gene, to a pool of 30 optimally-designed proprietary siRNAs from Biotech. The company does not disclose the sequences of these siRNAs.

Minor comments:

Point 6
Q. There needs to be an abbreviation section. Make sure that you only abbreviate the terms that are used more than once in the text.
A. An abbreviation list is now provided.

Point 7
Q. Lines 104, 277, 283 and anywhere else: use TG instead of thapsigargin.
A. Thank you for noting this. This has now been done.

Point 8
Q. Line 262: you don't have to redefine SERCA.
A. Done

Point 9
Q. I suggest adding structures of the used drugs.
A. The structures of the drugs used in this work are now presented in Figure S9.

Point 10
Q. I suggest using a table for the RT-PCR primers.
Protein Direction Number Sequence Description NCBI entry
h-SERCA2 Fwd #1612 5'ATG GGG CTC CAA CGA GTT AC nucleotides 648-667 of human SERCA2, variant a NM_001681.4
A. Thank you for this suggestion that we have now followed and that indeed facilitates the reading of the RT-PCR method section.

Point 11
Q. Line 93: DMEM (Gibco; ref 61965-059;) the lot number is missing.
A. The lot number is now indicated.

Point 12
Q. Line 102: 500'000 (and all other thousand numbers) the apostrophe's place is strange.
A. We have now removed the apostrophe in numbers.

Point 13
Q. Line 381: cholesterol carriers.
A. This typo has now been corrected

Reviewer #2 (Evidence, reproducibility and clarity (Required)):

Major concerns

Point 14
Q. 1. Figure 2, The authors should perform western blot to evaluate the protein expression levels (not only mRNA levels by Q-PCR)
A. We have performed these experiments in the past in MIN6 cells (Pétremand et al. Diabetes 2012 May; 61(5): 1100-1111; Figure 2). This earlier work showed that HDLs reduce the induction of TG-induced ER stress markers at the protein (CHOP and BiP) and functionality (IRE1 activity on XBP1 splicing). We will repeat these experiments in DLD1 cells as per the reviewer's suggestion.

© 2021. Published by The Company of Biologists under the terms of the Creative Commons Attribution License (https://creativecommons.org/licenses/by/4.0/).

Point 15.
Q. 2. Could the authors evaluate whether HDL treatment reduces the amount of SERCA (mRNA/protein) in their cells? The loss of SERCA could explain the reduced accumulation of the BODIPY-TG in the cell?
A. We would argue that it is unlikely that a reduction in SERCA expression from cells has any significant impact on TG cell loading as the cell-associated drug is certainly in vast excess compared to the number of SERCA molecules in cells. We will nevertheless perform the requested experiment using DLD-1 cells and assess whether HDLs modulate their SERCA2 expression.

Point 16.
Q. 3. To generalize their observation, It would have been interesting to test more lipophilic/hydrophilic drugs to quantitatively validate that HDLs are selective of lipophilic drugs.
A. We will test 2 new lipophilic (letermovir and lumefantrine) and 2 new hydrophilic drugs (levetiracetam and cefepime) for their ability to be extracted by HDLs (experiment set-up as in Figure 4).

Point 17.
Q. 4. The ABC transporter part in this manuscript has to be improved with the down-regulation of extinction of ABCA1 and ABCG1 to determine in a comprehensive manner the effect of these transporters in the pro-survival role of HDL.
A. We will invalidate the genes encoding ABCA1, ABCB1, ABCG1, and ABCG2 using the CRISPR/Cas9 technology and test the ability of the invalidated cells to promote efflux of thapsigargin to HDLs (experiment set-up as in Figure 6) and to protect them from the drug (experiment set-up as in Figure 6). The choice of the cell lines to be used for the invalidation depends on what ABC transporters they express. No single cell line expresses all four ABC transporters to high levels. The following cell lines will be used because, according to the literature or to the Human Protein Atlas (https://www.proteinatlas.org/), they display strong expression of the indicated transporters: for ABCA1: HCT116; for ABCB1: HEK293T; for ABCG1 and ABCG2: MCF7. For consistency with the experiments already performed in the manuscript, the invalidation will also be performed in the DLD1 cell line.

Minor point:

Point 18.
Q. 1. ABCB1 blot in figure 7B is not convincing and should be improved.
A. We will redo this WB to improve the quality of the blot.

Original submission

<u>First decision letter</u>

MS ID#: JOCES/2021/258644

MS TITLE: HDLs extract lipophilic drugs from cells

AUTHORS: Adi Zheng, Gilles Dubuis, Carla Susana Susana Mendes Ferreira, Thomas Mercier, Laurent Decosterd, and Christian Widmann
ARTICLE TYPE: Research Article

We have now reached a decision on the above manuscript.

To see the reviewers' reports and a copy of this decision letter, please go to: https://submit-jcs.biologists.org and click on the 'Manuscripts with Decisions' queue in the Author Area. (Corresponding author only has access to reviews.)

Thank you for submitting this interesting manuscript with reviews from Review Commons to the Journal of Cell Science. I have read the manuscript, the reviews, and your plans for revision in

© 2021. Published by The Company of Biologists under the terms of the Creative Commons Attribution License (https://creativecommons.org/licenses/by/4.0/).

response to the reviewer comments. In my opinion, you have outlined a reasonable set of experiments and these would be useful for addressing the reviewers' concerns. I agree that it seems unlikely that the HDLs are altering the levels of SERCA, though as you mention this is easy to test. Testing the additional lipophilic and hydrophilic drugs is also useful. Regardless, the results will be useful for understanding the specificity of HDL drug extraction. KO of the ABC transporters would provide definitive evidence for their role in this process. Perhaps, you could focus on one cell line (e.g. DLD1 cell line) and focus on the transporters that are highly expressed in this single cell line, rather than generating several KOs in multiple cell lines. This may shorten the time for the revisions. Is it necessary that these hydrophobic drugs, with diverse structures, employ a transporter?

If you able to execute the planned experiments to address the criticisms that were raised, I would be pleased to consider the revised manuscript.

We are aware that you may be experiencing disruption to the normal running of your lab that makes experimental revisions challenging. If it would be helpful, we encourage you to contact us to discuss your revision in greater detail. Please send us a point-by-point response indicating where you are able to address concerns raised (either experimentally or by changes to the text) and where you will not be able to do so within the normal timeframe of a revision. We will then provide further guidance. Please also note that we are happy to extend revision timeframes as necessary.

Please ensure that you clearly highlight all changes made in the revised manuscript. Please avoid using 'Tracked changes' in Word files as these are lost in PDF conversion.

I should be grateful if you would also provide a point-by-point response detailing how you have dealt with the points raised by the reviewers in the 'Response to Reviewers' box. Please attend to all of the reviewers' comments. If you do not agree with any of their criticisms or suggestions please explain clearly why this is so.

First revision

Author response to reviewers' comments

We thank both reviewers for their insightful comments and suggestions. We believe that addressing them has allowed improving our work.

Reviewer 1

Major points:

Point 1
Q. A logical question comes up and I do not think the authors addressed, in a human body what happens to the extracted drugs after loading on HDLs? This requires some mentioning in the discussion.
A. This is indeed a good question. We have now added in the discussion what may happen to the HDL- extracted drugs in a whole organism. It reads as follows:
The likely fate of HDL-extracted drugs in humans is that they are carried to the liver by HDLs. Scavenger receptors such as SR-BI expressed by hepatocytes can then bind HDLs carrying the extracted drugs allowing the drugs to be taken up by the cells. In hepatocytes, the drugs may be inactivated and excreted in the bile (https://doi.org/10.1016/j.cld.2016.08.001, https://doi.org/10.1161/CIRCRESAHA.119.312617).

Point 2
Q. Is the effect specific to the fully mature HDL molecule or do apo-lipoproteins that compose HDLs have similar effects?
A. This is an interesting question. Apo-AI is the characteristic and most abundant apolipoprotein

© 2021. Published by The Company of Biologists under the terms of the Creative Commons Attribution License (https://creativecommons.org/licenses/by/4.0/).

found in HDLs. It is however not trivial to compare the activities of ApoAI and HDLs because of the difficulty of producing large amounts of ApoAI. In the present paper, the lowest concentration of HDLs that induces drug efflux is 0.125 mM. As there are about 3 molecules of Apo-AI per HDL molecule, we should use 0.375 (3 x 0.125) mM Apo-AI to see if the Apo-AI content of these HDLs can mediate or mimic the drug efflux capacity of the lipoproteins. About 100 mg of recombinant Apo-AI would be required to make 10 ml of a ~0.3 mM Apo-AI cell culture solution. This is an enormous task requiring substantial time and money investment. We are therefore not in a position to perform this experiment that would be of interest but which is not central for supporting the main message of our manuscript.

Point 3
Q. What are non-SERCA-mediated effects of TG?
A. The SERCA-independent toxic effects of TG have been shown to be a consequence of mitochondrial dysfunction resulting from the ability of TG to induce mitochondrial permeability transition (DOI: 10.1046/j.1432-1327.1999.00724.x). This is now mentioned in the discussion.

Point 4
Q. Why don't HDLs protect cells from low dose TG despite its removal?
A. Our data indicate indeed that HDLs do not affect the ability of TG to inhibit SERCA and the low ER stress response that ensues. This can be explained by the fact that very low concentrations of TG inhibit SERCA in an irreversible manner (Ki values of 0.2, 1.3, and 12 nM for SERCA1b, SERCA2b, and SERCA3a, respectively) (DOI:https://doi.org/10.1074/jbc.M510978200). Hence, even though HDLs can remove a substantial amount of TG from cells, the concentration of TG that remains in cells is presumably still sufficient to fully inhibits the SERCA pumps. This explanation is now included in the discussion.

Point 5
Q. Line 144. No information on the siRNA was given (refer to the materials section to guide the reader).
A. The siPOOLs we have used correspond, for each targeted gene, to a pool of 30 optimally-designed proprietary siRNAs from Biotech. The company does not disclose the sequences of these siRNAs.

Minor comments:

Point 6

Q. There needs to be an abbreviation section. Make sure that you only abbreviate the terms that are used more than once in the text.
A. An abbreviation list is now provided.

Point 7
Q. Lines 104, 277, 283 and anywhere else: use TG instead of thapsigargin.
A. Thank you for noting this. This has now been done.

Point 8
Q. Line 262: you don't have to redefine SERCA.
A. Done

Point 9
Q. I suggest adding structures of the used drugs.
A. The structures of the drugs used in this work are now presented in Figure S8.

Point 10
Q. I suggest using a table for the RT-PCR primers.
Protein Direction Number Sequence Description NCBI entry
h-SERCA2 Fwd #1612 5'ATG GGG CTC CAA CGA GTT AC nucleotides 648-667 of human SERCA2, variant a NM_001681.4
A. Thank you for this suggestion that we have now followed and that indeed facilitates the reading of the RT-PCR method section (see Table 2).

© 2021. Published by The Company of Biologists under the terms of the Creative Commons Attribution License (https://creativecommons.org/licenses/by/4.0/).

Point 11
Q. Line 93: DMEM (Gibco; ref 61965-059;) the lot number is missing.
A. The lot number is now indicated.
Point 12
Q. Line 102: 500'000 (and all other thousand numbers) the apostrophe's place is strange.
A. We have now removed the apostrophe in numbers.

Point 13
Q. Line 381: cholesterol carriers.
A. This typo has now been corrected

Reviewer #2 (Evidence, reproducibility and clarity (Required)):

Major concerns

Point 14
Q. 1. Figure 2, The authors should perform western blot to evaluate the protein expression levels (not only mRNA levels by Q-PCR)
A. We have performed these experiments in the past in MIN6 cells (Pétremand et al. Diabetes 2012 May; 61(5): 1100-1111; Figure 2). This earlier work showed that HDLs reduce the induction of TG- induced ER stress markers at the protein (CHOP and BiP) and functionality (IRE1 activity on XBP1 splicing). We have now repeated these experiments in DLD1 cells as per the reviewer's suggestion (see updated Figure 2). Our results show that both CHOP expression at the protein level and XBP1 splicing induced by TG are inhibited by HDLs.

Point 15.
Q. 2. Could the authors evaluate whether HDL treatment reduces the amount of SERCA (mRNA/protein) in their cells? The loss of SERCA could explain the reduced accumulation of the BODIPY-TG in the cell?
A. We would argue that it is unlikely that a reduction in SERCA expression from cells has any significant impact on TG cell loading as the cell-associated drug is certainly in vast excess compared to the number of SERCA molecules in cells. We have nevertheless performed the requested experiment using DLD-1 cells. New Figure S6 shows that HDLs do not modulate SERCA2 expression.

Point 16.
Q. 3. To generalize their observation, It would have been interesting to test more lipophilic/hydrophilic drugs to quantitatively validate that HDLs are selective of lipophilic drugs.
A. We have tested 2 new lipophilic (letermovir and lumefantrine) and 1 new hydrophilic compound (FITC-D-TAT) for their ability to be extracted by HDLs (see updated Figures 4 and 6). HDLs were found to extract the letermovir and lumefantrine drugs but they had no impact on FITC-D-TAT release from cells.

Point 17.
Q. 4. The ABC transporter part in this manuscript has to be improved with the down-regulation of extinction of ABCA1 and ABCG1 to determine in a comprehensive manner the effect of these transporters in the pro-survival role of HDL.
A. To assess the involvement of four ABC transporter (ABCA1, ABCB1, ABCG1, and ABCG2) previously suggested to mediate the cellular efflux of xenobiotics, we chose DLD1 cells that do not express ABCA1 and ABCG2 (Figure S6A) and, using the CRISPR/Cas9 technology, we invalidated in these cells the genes encoding ABCB1 and ABCG1 (Figure S7A-B). The resulting DLD1 ABCB1/ABCG1 double knock-out cells therefore lack ABCA1, ABCB1, ABCG1, and ABCG2. Thapsigargin was efficiently extracted by HDLs in both the double knock-out and in the wild-type parental DLD1 cells (Figure S7C). These data indicate that even though specific ABC transporters (e.g. ABCB1) may favor HDL-mediated drug extraction in some cell lines (e.g. HEK293T cells; see Figure 8), ABC transporters are not necessarily required for efficient HDL-mediated drug efflux

© 2021. Published by The Company of Biologists under the terms of the Creative Commons Attribution License (https://creativecommons.org/licenses/by/4.0/).

Minor point:
Point 18.
Q. 1. ABCB1 blot in figure 7B is not convincing and should be improved.
A. We have now redone this blot. Please see new Figure 8B (reproduced here).

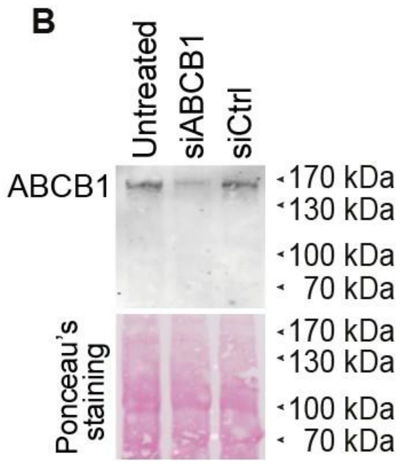

<u>Second decision letter</u>

MS ID#: JOCES/2021/258644

MS TITLE: HDLs extract lipophilic drugs from cells

AUTHORS: Adi Zheng, Gilles Dubuis, Maria Georgieva, Carla Susana Susana Mendes Ferreira, Marc Serulla, Maria del Carmen Conde Rubio, Evgeniya Trofimenko, Thomas Mercier, Laurent Decosterd, and Christian Widmann
ARTICLE TYPE: Research Article

Thank you for sending your manuscript to Journal of Cell Science through Review Commons.

I am happy to tell you that your manuscript has been accepted for publication in Journal of Cell Science, pending standard ethics checks. Thank you for your thoughtful responses to the referee comments and for submitting this interesting work to JCS!

© 2021. Published by The Company of Biologists under the terms of the Creative Commons Attribution License (https://creativecommons.org/licenses/by/4.0/).
